# Complexity of progranulin mechanisms of action in mesothelioma

**DOI:** 10.1186/s13046-022-02546-4

**Published:** 2022-12-05

**Authors:** Elisa Ventura, Christopher Xie, Simone Buraschi, Antonino Belfiore, Renato V. Iozzo, Antonio Giordano, Andrea Morrione

**Affiliations:** 1 Sbarro Institute for Cancer Research and Molecular Medicine, Center for Biotechnology, Department of Biology, College of Science and Technology, Temple University, Philadelphia, PA 19122 USA; 2 Department of Pathology, Anatomy and Cell Biology, Translational Cellular Oncology Program, Sidney Kimmel Cancer Center, Sidney Kimmel Medical College at Thomas Jefferson University, Philadelphia, PA 19107 USA; 3 Department of Clinical and Experimental Medicine, Endocrinology Unit, University of Catania, Garibaldi-Nesima Hospital, 95122 Catania, Italy; 4 Department of Medical Biotechnologies, University of Siena, 53100 Siena, Italy

**Keywords:** Mesothelioma, Progranulin, EphA2, EGFR, RYK, FAK, Migration, Invasion, Focal adhesion turnover

## Abstract

**Background:**

Mesothelioma is an aggressive disease with limited therapeutic options. The growth factor progranulin plays a critical role in several cancer models, where it regulates tumor initiation and progression. Recent data from our laboratories have demonstrated that progranulin and its receptor, EphA2, constitute an oncogenic pathway in bladder cancer by promoting motility, invasion and *in vivo* tumor formation. Progranulin and EphA2 are expressed in mesothelioma cells but their mechanisms of action are not well defined. In addition, there are no data establishing whether the progranulin/EphA2 axis is tumorigenic for mesothelioma cells.

**Methods:**

The expression of progranulin in various mesothelioma cell lines derived from all major mesothelioma subtypes was examined by western blots on cell lysates, conditioned media and ELISA assays. The biological roles of progranulin, EphA2, EGFR, RYK and FAK were assessed *in vitro* by immunoblots, human phospho-RTK antibody arrays, pharmacological (specific inhibitors) and genetic (siRNAs, shRNAs, CRISPR/Cas9) approaches, motility, invasion and adhesion assays. *In vivo* tumorigenesis was determined by xenograft models. Focal adhesion turnover was evaluated biochemically using focal adhesion assembly/disassembly assays and immunofluorescence analysis with focal adhesion-specific markers.

**Results:**

In the present study we show that progranulin is upregulated in various mesothelioma cell lines covering all mesothelioma subtypes and is an important regulator of motility, invasion, adhesion and *in vivo* tumor formation. However, our results indicate that EphA2 is not the major functional receptor for progranulin in mesothelioma cells, where progranulin activates a complex signaling network including EGFR and RYK. We further characterized progranulin mechanisms of action and demonstrated that progranulin, by modulating FAK activity, regulates the kinetic of focal adhesion disassembly, a critical step for cell motility.

**Conclusion:**

Collectively, our results highlight the complexity of progranulin oncogenic signaling in mesothelioma, where progranulin modulate functional cross-talks between multiple RTKs, thereby suggesting the need for combinatorial therapeutic approaches to improve treatments of this aggressive disease.

**Supplementary Information:**

The online version contains supplementary material available at 10.1186/s13046-022-02546-4.

## Background

Malignant mesothelioma (MM), is an aggressive tumor with a median survival of 12 months [1]. A major fraction of mesothelioma cases is linked to asbestos exposure, with a long latency period of 20–50 years between exposure and mesothelioma development [2]. As a consequence of asbestos ban in Europe and some other countries in the’80 and’90, we are observing a decline in the percentage of mesothelioma patients who have been exposed to asbestos [3, 4]. Indeed, asbestos-related mesothelioma are mainly seen in older patients who were exposed to asbestos before the introduction of the ban. However, there is an increase in mesothelioma cases not associated with asbestos exposure mostly affecting younger patients. Around 12% of patients presenting with asbestos-unrelated mesotheliomas carries germline mutations in BRCA1-associated protein 1 (BAP1) or other tumor suppressor genes [3, 4].

Multiple novel targets and pathways of interest have been identified from genomic studies of MM [5]. However, in MM, mutations do not usually affect growth-regulating kinases as in many other tumor types, rather they affect tumor suppressor genes, whose targeting is more complex. In addition, there are no predictive biomarkers of therapy response [5]. There are three major histologic subtypes of MM, epithelioid, biphasic and sarcomatoid with different prognosis [6]. However, a more detailed subclassification and histologic/cytological characterization of MM might have prognostic and perhaps predictive value for MM [6, 7]. In spite of considerable work done in recent years to develop immunotherapies and biomarker-driven therapy to improve patient outcomes, there is still an unmet need for the identification of novel targets to improve therapy.

Progranulin is a pluripotent growth factor containing 7 and a half highly-conserved granulins [8] cleaved by elastase and MMPs to produce granulin peptides A-G and paragranulin (p) [9, 10]. Progranulin is implicated in several human diseases including cancer, neurodegenerative diseases and rheumatoid arthritis [11–13]. We have established that progranulin plays a critical role in prostate [14–17] and bladder cancer by promoting tumor cell motility and invasion [18–21], *in vivo* tumor growth and sensitizing cancer cells to cisplatin treatment [22]. We recently provided a significant advance in the field by identifying a functional membrane receptor for progranulin, EphA2 [23], and later demonstrated that the progranulin/EphA2 is a critical oncogenic axis in bladder cancer [24]. The Eph receptors constitute the largest family of receptor tyrosine-kinase (RTKs) and are important regulators of development and disease [25–27]. EphA2 activation by its natural ligand ephrinA1 (*canonical signaling*) regulates cellular repulsion and adhesion, but the role of EphA2 in cancer is more complex with data suggesting either pro- or anti-oncogenic functions [28]. For example, in the presence of ephrinA1, EphA2 is dephosphorylated at S897 leading to inhibition of cancer cell motility and invasion. Conversely, ephrinA1-independent AKT or RSK activation (*non-canonical signaling*) evokes EphA2 phosphorylation at S897 enhancing EphA2 oncogenic activity [29].

Progranulin is expressed in mesothelioma cells and constitutes a VEGF-independent angiogenic factor [30]. Significantly, EphA2 is expressed in MM where it is either overexpressed, mutated or amplified [31]. Notably, the role of EphA2 in MM is still controversial and data suggest either a positive or negative role in modulating MM transformation. EphA2 activation with the ligand ephrinA1 inhibits growth of MM [32] indicating that ephrinA1-dependent EphA2 action has likely a tumor suppressive function in mesothelioma. On the contrary, transient EphA2 depletion by siRNA approaches inhibited growth and haptotaxis of MM cells, but the experimental conditions were not clearly defined [33]. However, there are no data connecting progranulin tumorigenic action with *non-canonical* (ephrinA1-independent) EphA2 activation in MM and therefore no evidence that the progranulin/EphA2 axis might be activated and constitute an oncogenic pathway in MM.

Here we demonstrated that progranulin is critical for the regulation of motility, invasion, adhesion and *in vivo* tumor growth of MM cells by modulating FAK activity and focal adhesion turnover (assembly/disassembly). However, EphA2 is not the major signaling receptor for progranulin in MM cells, where progranulin relies on the activation of EGFR and RYK, suggesting that in MM the progranulin axis modulates a complex network of RTKs signaling, which might constitute novel targets for therapy.

## Materials and methods

### Cell culture and reagents

MeT-5A, NCI-H2052, NCI-H2452, NCI-H28 and MSTO-211H cells were provided by ATCC and cultured in RPMI (Thermo Scientific, Waltham, MA, USA), supplemented with 10% FBS (R&D Systems, Minneapolis, MN, USA) and 1% L-glutamine (Thermo Scientific Scientific).

The MEK1/2 inhibitor U0126, the FAK inhibitor PF-573228, the EGFR inhibitor gefitinib and nocodazole were from Cayman Chemicals (Ann Arbor, MI, USA) and the AKT inhibitor AKTi VIII form Calbiochem (San Diego, CA, USA). His-tag human recombinant progranulin was prepared as previously described [34].

### Gene depletion and expression

Transient gene depletion was obtained by transfecting cells with ON-TARGET plus small interfering RNAs (siRNA) from Dharmacon (Lafayette, CO, USA) targeting *GRN* (progranulin) (L-009285–00-0005), *EphA2* (L-003116–00-0005), *PTK2* (FAK) (L-003164–00-0005), *EphA7* (L-003119–00-0005), *RYK* (L-003174–00-0005) or non-targeting control siRNA (D-001810–10-05), using the Dharmafect transfection reagent according to the Manufacturer’s instruction. siRNAs targeting progranulin, *PTK2* and *EphA7* were used at the final concentration of 25 nM, siRNAs specific for *EphA2* and *RYK* at 50 nM.

MSTO-211H and NCI-H2052 cells with progranulin or *EphA2* knock-out were generated using CRISPR/Cas9 strategies as previously described [35]. The sgRNAs GGTGGCCTTAACAGCAGGGC [36] and GAAGCGCGGCATGGAGCTCC targeting *GRN* and *EphA2* were used respectively.

Stable gene silencing of *RYK* was achieved using RYK-specific short hairpin RNAs (shRNA) (V2LHS_31986 and V3LHS_345296, Dharmacon) and non-targeting control shRNA (RHS4346), cloned in a pGIPZ lentiviral vector (Dharmacon). pGIPZ vectors were used to generate lentivirus and transduce cells as previously described [35].

To prepare *GRN* KO MSTO-211H cells with reconstituted expression of progranulin and progranulin overexpressing NCI-H2052 cells, cDNA coding for human progranulin was cloned into the lentiviral vector pLenti CMV Puro DEST as previously described [35]. Human progranulin cDNA was amplified by PCR using Phusion high-fidelity DNA polymerase (New England Biolabs, Ipswich, MA, USA), the primer pair *GRN* fwd/*GRN* rev (Supplementary table 1) and total cDNA derived from MSTO-211H cells as DNA template. MSTO-211H-derived total cDNA was prepared by retrotranscribing 1 µg of total RNA extracted from MSTO-211H cells using the Applied Biosystems High-Capacity cDNA Reverse transcription kit (Thermo Fisher Scientific). pLenti CMV Puro DEST containing the progranulin sequence was used to prepare lentiviral particles and transduce cells as previously described [35].

To reconstitute EphA2 expression in *EphA2* KO MSTO-211H cells, we used the retroviral plasmid pCLXSN-EphA2-Flag, a gift from Jin Chen (Addgene plasmid #102755, Addgene, Wartertown, MA, USA). EphA2 mutants were generated using Phusion high-fidelity DNA polymerase (New England Biolabs), the primers reported in Supplementary table 1 and plasmid pCLXSN-EphA2-Flag as DNA template. To generate retroviral particles expressing wild type or mutants EphA2, HEK-293FT cells were transfected with the pMD2-G envelope plasmid, the pUMVC packaging plasmid (a gift of Bob Weinberg, Addgene plasmid #8449) and the pCLXSN retroviral vectors containing the cDNA coding for wild type or mutants EphA2. HEK-293FT-transfected conditioned media supplemented with 8 µg/ml polybrene (Sigma-Aldrich) was used to transduce *EphA2* KO MSTO-211H cells as described [37, 38].

### Migration and invasion assays

Cell migration was assessed by transwell migration assays using 8.0 µm pore polyester membrane inserts (Corning, Glendale, AZ, USA). Serum-starved cells were seeded in SFM in the upper chamber at a cell density of 3 × 10^4^ (MSTO-211H cells) or of 2 × 10^4^ (NCI-H2052 cells). The lower chamber was filled with 5% FBS-supplemented medium (MSTO-211H) or SFM with or without 50 nM progranulin (NCI-H2052). After 30 h (MSTO-211H) or 16 h (NCI-H2052), cells on the filter upper surface were removed with a cotton swab while cells on the filter lower surface were fixed with ice-cold methanol, stained with Coomassie Brilliant Blue and counted under a DMi1 inverted microscope (Leica, Wetzlar, Germany). Cell invasion through a three-dimensional extracellular matrix was measured using inserts containing an 8 µm pore-size membrane with a uniform layer of matrigel matrix (Corning). Experiments were performed as described for migration but cells were allowed to invade for 40 h (MSTO-211H) or 24 h (NCI-H2052).

### Adhesion assay

For adhesion assay, 24-well plates were coated over-night with 5 µg/ml plasma fibronectin (R&D Systems) in PBS, a collagen coating solution (Sigma Aldrich, St. louis, MO, USA) or 0.01% Poly-L-Lys (Cultrex Poly-L-Lysine, R&D Systems), washed with PBS and blocked with 2% BSA in PBS. 2% BSA-coated wells were used as a negative control. Serum-starved cells were detached, and conditioned media collected and aliquoted. Cells were then resuspended in an aliquot of their respective conditioned media supplemented with 2.5 µM Calcein AM fluorescent dye (Cayman Chemicals) and incubated at 37 °C for 30 min. Cells were then centrifuged, resuspended in another aliquot of conditioned medium and seeded at a cell density of 0,15  × 10^6^ cells/well on fibronectin-, collagen- or Poly-L-Lys-coated wells. Cells were allowed to adhere for 30 min (MSTO-211H) or 20 min (NCI-H2052) at 37 °C, 5% CO2. Fluoresce intensity at λex = 494 nm and λem = 517 nm was then measured using a Victor5 plate reader (Perkin Elmer, Waltham, MA, USA). Plates were then washed two (MSTO-211H) or three (NCI-H2052) times with PBS and fluorescence intensity was measured again. The fraction of cells that adhered to the various substrates was determined dividing the fluorescence intensity recorded after plate wash by the fluorescence intensity measured before plate wash. Similarly, the minimal fraction of cells that adhered to BSA was determined and used to normalize the data. Cell–cell adhesion was assessed in a similar way, by seeding cells stained with Calcein AM on parental, PGRN-overexpressing or *EphA2* KO NCI-H2052 cell monolayers.

### *In vivo* xenograft

*In vivo* experiments were performed according to protocols approved by the Institutional Review Board of Thomas Jefferson University. Eight-to-twelve-weeks-old *Rag*2-/- mice were subcutaneously implanted in the flank with 4 × 10^6^ MSTO-211H parental, *GRN* KO, and *EphA2* KO cells. Tumor volumes were measured every two-days using a micro-caliper and the following formula: V = a(b^2^/2). When tumors reached a volume of 1500 mm^3^, mice were sacrificed.

### Immunoblot

Cell lysates were prepared using RIPA buffer (Thermo Fisher Scientific) supplemented with halt protease and phosphatase inhibitors cocktail (Thermo Fisher Scientific). The following primary antibodies were used for immunoblot analysis: pEphA2 S897 (6347), EphA2 (6997), pAKT S473 (4060), pan-AKT (4691), pERK1/2 (4370), ERK1/2 (9102), pFAK Y397 (3285), FAK (8556), pEGFR Y1068 (3777), EGFR (4267), and EphA7 (64,801) from Cell Signaling Technology (Danvers, MA, USA), progranulin (P-9182-35B, US Biologicals, Salem, MA, USA), pEphA2 S901 (PA5-105,552, Thermo Scientific), and GAPDH (sc-365062) (Santa Cruz Biotechnology, Dallas, TX, USA). The following secondary antibodies were used: anti-rabbit HRP-linked (7074) (Cell Signaling Technology) and the antibodies from Santa Cruz Biotechnology mouse anti-rabbit IgG-HRP (sc-2357) and m-IgGk BP-HRP (sc-516102).

Immunoblots were quantified using the ImageJ program and expressed as arbitrary units (AU).

### Enzyme-linked immunosorbent assay

Progranulin concentration was measured in cell conditioned media using the human progranulin quantikine ELISA kit (R&D Systems) following the Manufacturer’s instructions. Protein concentration was normalized to total cell number.

### Phospho-RTK array

Human phospho-RTK antibody arrays membranes (R&D Systems) were incubated with 0.7 mg of cell lysates from MSTO-211H or NCI-H2052 cells serum-starved for 24 h, untreated or stimulated with 10 nM progranulin for 15 min and processed according to the manufacturer’s instructions.

The intensity of the dots was quantified using the ImageJ program.

### Immunofluorescence analysis

For immunofluorescence experiments cells were washed with PBS, fixed with 4% PFA in PBS for 15 min, permeabilized with 0.1% triton X-100 in PBS for 10 min and blocked with 2% BSA in PBS for 1 h at RT. Fixed cells were incubated over-night at 4 °C with the following primary antibodies diluted in 2% BSA: pFAK (Y397) (44-624G) (Thermo Fisher Scientific) and the monoclonal anti-vinculin antibody clone hVIN-1 (NB-600–1293) (Novus Biologicals, Centennial, CO, USA). Secondary antibodies were goat-anti rabbit IgG (H + L) Alexa Fluor-488 and goat anti mouse IgG (H + L) Alexa Fluor 555 (Thermo Fisher Scientific). F-actin was stained using the Phalloidin-iFluor 488 reagent (Abcam, Cambridge, UK). Nuclei were stained using the *SlowFade* Gold Antifade Mountant with DAPI (Thermo Fisher Scientific). Images were acquired using an Olympus IX81 fluorescent microscope (Olympus, Tokyo, Japan) equipped with a Retiga 6000 camera (QImaging, Surrey, Canada) and the Olympus cellSens program (Olympus).

### RT-PCR

RT-PCR was performed using the delta-CT method [39] using β-actin as a housekeeping gene. Primers specific for RYK and β-actin are reported in Supplementary Table 1.

### Statistical analysis

Data are shown as mean ± the standard deviation (s.d.). Statistical significance was determined by performing one-way ANOVA followed by Tukey's post hoc test at 95% confidence interval (CI) or the Student t-test. A *p*-value < 0.05 was considered statistically significant. In ELISA, migration, invasion and adhesion assays and qPCR experiments samples were analyzed in triplicates. *In vitro* experiments were repeated three times.

## Results

### Progranulin promotes AKT and MAPK activation and EphA2 phosphorylation at S897 in mesothelioma cells

**Fig. 1:**
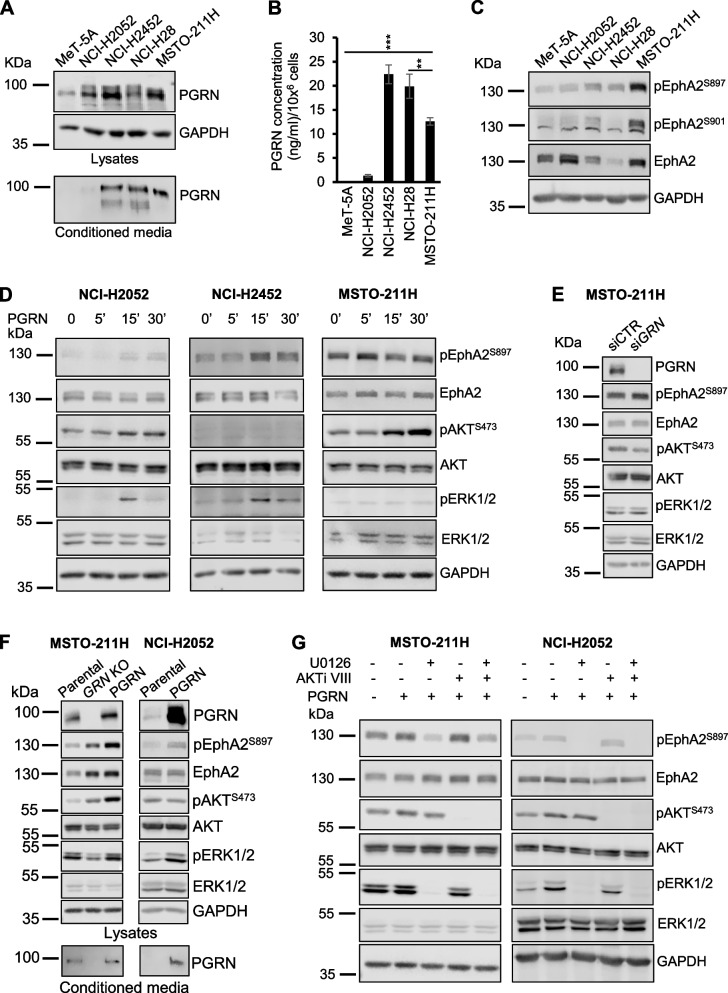
Progranulin promotes AKT and MAPK activation in mesothelioma cells. **A** Progranulin (PGRN) protein levels were analyzed by immunoblot in cell lysates and media conditioned from MeT-5A, NCI-H2052, NCI-H2452, NCI-H28 and MSTO-211H cells. **B** Progranulin levels in media conditioned from MeT-5A, NCI-H2052, NCI-H2452, NCI-H28 and MSTO-211H cells was measured by ELISA assay. *N* = 3, ± SD, ** *p* < 0.01, *** *p* < 0.001. **C** Levels of total and phosphorylated EphA2 (S897 and S901) were analyzed by immunoblot in cell lysates derived from MeT-5A, NCI-H2052, NCI-H2452, NCI-H28 and MSTO-211H cells serum-starved for 24 h. **D** Levels of total and phosphorylated EphA2 (S897), AKT and ERK1/2 were assessed by immunoblot in NCI-H2052, NCI-H2452 and MSTO-211H cells serum-starved for 24 h and then treated with 50 nM progranulin for the indicated time. **E** MSTO-211H cells were transfected with siRNA targeting progranulin or non-targeting control siRNA. At 8 h post-transfection cells were transferred into serum-free medium for 40 h and then analyzed by immunoblot for total and phosphorylated levels of EphA2 (S897), AKT, ERK1/2 and progranulin levels. **F** Levels of total and phosphorylated EphA2, AKT, ERK1/2 and progranulin were assessed by immunoblot in parental MSTO-211H, MSTO-211H cells with progranulin knock-out by CRISPR/Cas9 (*GRN* KO) and *GRN* KO MSTO-211H cells with reconstituted progranulin expression (left panel) or parental and progranulin overexpressing NCI-H2052 cells (right panel), serum-starved for 24 h. Progranulin levels were also analyzed in conditioned media from cells starved for 48 h. **G** MSTO-211H and NCI-H2052 cells were serum-starved for 24 h, pre-incubated with either the MEK1/2 inhibitor U0126 (10 µM) or the AKT inhibitor AKTi VIII (5 µM) for 2 h and then treated with the same concentrations of MEK1/2 and AKT inhibitors alone or in combination with 50 nM progranulin for 15 min. Levels of total and phosphorylated EphA2, AKT and ERK1/2 were determined by immunoblot

To characterize the role of the progranulin/EphA2 axis in mesothelioma, we initially analyzed the expression levels of progranulin in a panel of mesothelioma cell lines representative of the three major histologic subtypes, sarcomatoid (NCI-H2052, NCI-H2452), epithelioid (NCI-H28) and biphasic (MSTO-211H). Progranulin was upregulated in all mesothelioma cell lines as compared to immortalized cells derived from normal mesothelium (MeT-5A), with the highest expression levels in NCI-H2452, NCI-H28 and MSTO-211H, as assessed by immunoblot in cell lysates, conditioned media and ELISA assay (Fig. 1A-B). We then investigated the expression levels of EphA2 and its phosphorylation at S897 and S901 [24]. All mesothelioma cell lines showed EphA2 phosphorylation at S897 above the level of MeT-5A cells, with MSTO-211H cells showing the highest levels, while EphA2 S901 phosphorylation was only detectable in NCI-H2452 and MSTO-211H cells (Fig. 1C). For our experiments we therefore focused on sarcomatoid and biphasic cells, considering that epithelioid NCI-H28 cells express very low levels of EphA2.

Next, we analyzed the signaling pathways activated by progranulin in mesothelioma cells. Progranulin stimulation promoted the activation of the AKT and MAPK pathways, with a more pronounced effect on the MAPK pathway in NCI-H2052 and NCI-H2452, while the AKT pathway was preferentially activated in MSTO-211H cells (Fig. 1D). These results suggest that progranulin signaling might slightly differ in mesothelioma cells originated from different mesothelioma subtypes. We also confirmed that progranulin promoted EphA2 phosphorylation at S897 in NCI-H2052, NCI-H2452 and MSTO-211H cells (Fig. 1D and Supplementary Fig. 4A). To assess whether AKT and ERK signaling was depending on endogenous progranulin, we used siRNA strategies and transiently depleted endogenous progranulin in MSTO-211H cells, which express the highest levels and show strong serine-phosphorylation of EphA2 (Fig. 1A-C). We achieved significant progranulin depletion, which was associated with a strong inhibition of AKT activation (Fig. 1E and Supplementary Fig. 4B), confirming therefore that AKT activation is, at least in part, dependent on progranulin autocrine signaling in these cells. To confirm these data, we generated MSTO-211H cells with *GRN* gene deletion by CRISPR/Cas9 approaches. *GRN* deleted cells showed increased levels of pAKT and pEphA2 S897 and decreased levels of pERK1/2 when compared to parental cells (Fig. 1F, left panel, and Supplementary Fig. 4C). The increased activation of AKT and EphA2 in *GRN* KO cells was unexpected and suggest that upon progranulin deletion AKT and EphA2 activation might be sustained by compensatory mechanisms. Significantly, restoring progranulin expression in *GRN* KO cells further increased the levels of pAKT and pEphA2 S897 and restored basal activation of ERK1/2 observed in parental MSTO-211H cells, thereby confirming a role for progranulin in modulating the activation of the AKT and MAPK pathways in MSTO-211H cells (Fig. 1F, left panel, and Supplementary Fig. 4C). To complement the *GRN* deletion data, we overexpressed progranulin in NCI-H2052 cells, which express low levels of endogenous progranulin (Fig. 1A-B) and tested AKT and MAPK activation. Progranulin-overexpressing cells showed increased pERK1/2 and pEphA2 S897 levels when compared to parental NCI-H2052 (Fig. 1F, right panel, and Supplementary Fig. 4C), further suggesting the role of progranulin in promoting the activation of MAPK and EphA2. Finally, we demonstrated that progranulin-induced EphA2 phosphorylation at S897 in MSTO-211H and NCI-H2052 cells was mediated by progranulin-dependent ERK1/2 activation, and to a lesser extent of AKT activation, as demonstrated by using specific pharmacological inhibitors of the AKT and MAPK pathways (Fig. 1G).

### Progranulin modulates cell motility, adhesion and *in vivo* tumor growth

To further decipher the contribution of EphA2 in modulating the progranulin axis in mesothelioma, we generated MSTO-211H and NCI-H2052 cells with genetic deletion of *EphA2* by CRISPR/Cas9 technology (Supplementary Fig. 1A-B) and compared the ability of *GRN*- and *EphA2*-KO cells to migrate and invade through matrigel [14, 18–20, 22, 24].

**Fig. 2:**
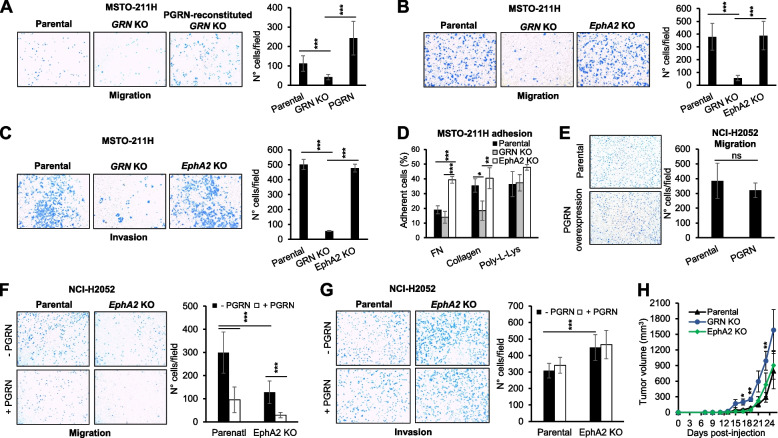
Progranulin and EphA2 differ in their ability to modulate mesothelioma cell motility and adhesion. **A** Migration of parental, *GRN* KO and progranulin-re-expressing *GRN* KO MSTO-211H cells was assessed using transwells as described in Material and Methods. **B** Migration of parental, *GRN* KO and *EphA2* KO MSTO-211H cells as assessed by transwells migration. **C** Invasion of parental, *GRN* KO and *EphA2* KO MSTO-211H cells was assessed using matrigel-coated transwells. **D** The ability of parental, *GRN* KO and *EphA2* KO MSTO-211H cells to adhere to plasma fibronectin, collagen and poly-L-Lys was assessed as described in Material and Methods. **E** The migratory ability of parental and progranulin overexpressing NCI-H2052 cells was assessed using transwells. **F** Transwell migration of parental and *EphA2* KO NCI-H2052 cells, untreated or stimulated with 50 nM progranulin. **G** Invasion through matrigel-coated transwells of parental and *EphA2* KO NCI-H2052 cells, untreated or stimulated with 50 nM progranulin. For motility and adhesion assays, data are the average of three independent experiments ± SD. *** *p* < 0.001, ** *p* < 0.01, * *p* < 0.05. **H** Parental, *GRN* KO and *EphA2* KO MSTO-211H cells were subcutaneously implanted in *Rag*2-/- mice and tumor volumes measured at the indicated time post tumor injection. *N* = 8, ± SD, * *p* < 0.05, ** *p* < 0.01

*GRN* KO MSTO-211H cells showed significantly reduced migratory ability as compared to parental MSTO-211H cells (Fig. 2A). Notably, progranulin expression in *GRN* KO cells restored their migratory capacity (Fig. 2A) indicating a specific role of progranulin in modulating MSTO-211H cells motility. Interestingly, parental and *EphA2* KO MSTO-211H cells migrated to a similar extent, suggesting that EphA2 loss did not significantly affect MSTO-211H cell motility (Fig. 2B). In addition, *GRN* KO MSTO-211H cells showed significantly impaired invasive capacity through matrigel as compared to MSTO-211H cells, whereas parental and *EphA2* KO MSTO-211H cells were similar in their invasive abilities (Fig. 2C). These results strongly suggest that progranulin regulates MSTO-211H cell motility independently of EphA2 activation. Based on these results suggesting a different action of progranulin and EphA2 in regulating MSTO-211H cell motility, we then assessed cell adhesion and evaluated the ability of parental, *GRN* and *EphA2* KO MSTO-211H cells to adhere to different substrates, including plasma fibronectin, collagen and poly-L-Lys. As shown in Fig. 2D, *GRN* KO cells showed a significantly reduced capacity to adhere to collagen when compared to parental cells (Fig. 2D), whereas *GRN* KO and parental MSTO-211H cells showed similar adhesion to plasma fibronectin and poly-L-Lys (Fig. 2D). By contrast, *EphA2* KO MSTO-211H cells showed a significant increased capacity to adhere to plasma fibronectin when compared to both parental and *GRN* KO cells (Fig. 2D). All together these results suggest that progranulin and EphA2 differ in their ability to modulate MSTO-211H adhesive properties.

Next, we investigated progranulin and EphA2 action in NCI-H2052 cell motility and adhesion. To this end, we first stably transfected NCI-H2052 cells with a plasmid expressing human progranulin and then compared the migratory ability of parental and progranulin-overexpressing cells. As shown in Fig. 2E, progranulin overexpression did not significantly affect the migration of NCI-H2052 (Fig. 2E). We subsequently investigated whether exogenous progranulin might modulate NCI-H2052 cell migration. Surprisingly, progranulin-stimulated NCI-H2052 cells showed a marked reduction in their migratory ability when compared to unstimulated cells (Fig. 2F). Next, we tested cell migration of parental and *EphA2* KO NCI-H2052 cells. As shown in Fig. 2F, *EphA2* KO cells showed a reduced migratory capacity in respect to parental cells and their migration was further reduced by recombinant progranulin (Fig. 2F). By contrast, recombinant progranulin did not affect parental and *EphA2* KO NCI-H2052 cells invasive abilities but *EphA2* KO cells showed increased basal (untreated cells) invasiveness when compared to parental cells (Fig. 2G). Based on these results, we then hypothesized that, in this cell line, progranulin might preferentially modulate cell adhesion. However, neither progranulin overexpression nor EphA2 deletion affected NCI-H2052 cell adhesion on collagen, plasma fibronectin and poly-L-Lys or interfered with cell–cell adhesion (Supplementary Fig. 2).

Finally, we compared the ability of parental, *GRN* or *EphA2* KO MSTO-211H cells to modulate *in vivo* tumor formation. Cells were subcutaneously implanted in the flank of *Rag2* ^−^/^−^ mice and tumors were monitored until they reached a volume of 1500 mm^3^. All cells generated tumor xenografts, with parental and *EphA2* KO MSTO-211H cells showing similar *in vivo* tumor formation (Fig. 2H). Notably, *GRN* KO cells generated tumors with significantly higher tumor volume as compared to both parental and *EphA2* KO MSTO-211H cells (Fig. 2H). These results suggest that EphA2 deletion is not critical for *in vivo* tumor formation of MSTO-211H cells while progranulin might play a more relevant role in modulating *in vivo* xenograft tumors.

### Progranulin-dependent activation of AKT and MAPK pathways does not require EphA2 in mesothelioma

**Fig. 3:**
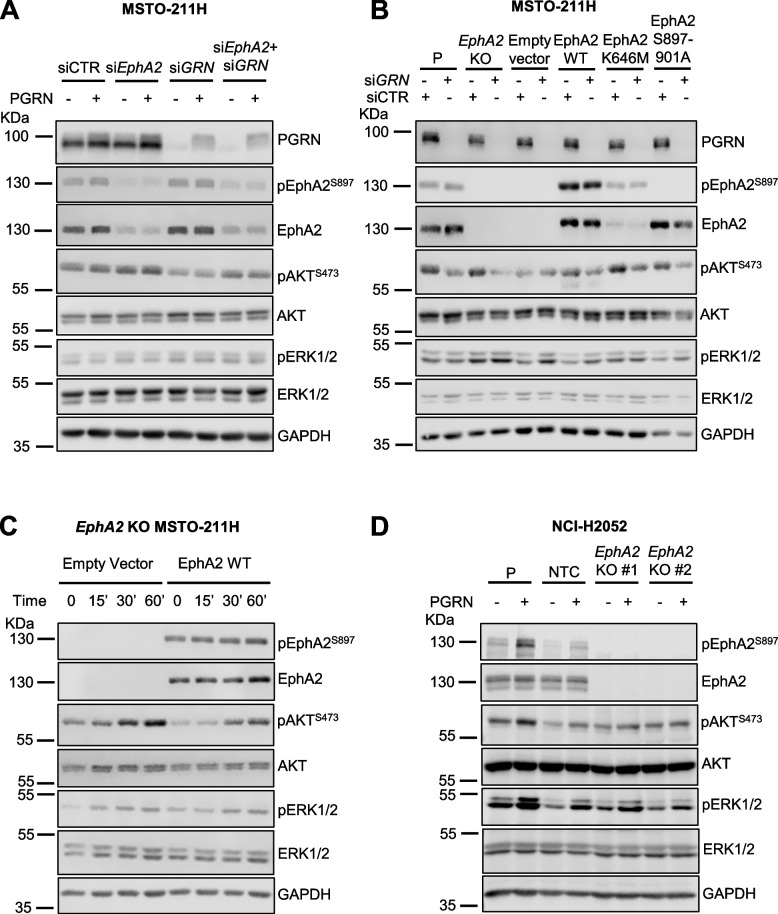
Progranulin activates AKT and ERK1/2 in an EphA2-independent manner in mesothelioma cells. **A** MSTO-211H cells were transfected with siRNA targeting either *GRN* (progranulin), *EphA2*, the combination of the two or non-targeting control (siCTR). At 8 h post-transfection cells were transferred to serum-free media and incubated for additional 40 h. Cells were then treated with 50 nM progranulin for 60 min. Levels of progranulin, total and phosphorylated EphA2 (S897), AKT and ERK1/2 were analyzed by immunoblot. **B** *GRN* was depleted using siRNAs targeting progranulin as described in (**A**) in parental MSTO-211H (P), *EphA2* KO MSTO-211H and *EphA2* KO cells re-expressing wild type EphA2 or the EphA2 mutants EphA2 K646M and EphA2 S897A/S899A/S901A. Levels of progranulin, total and phosphorylated EphA2 (S897), AKT and ERK1/2 were determined by immunoblot. **C** Total and phosphorylated EphA2 (S897), AKT and ERK1/2 as assessed by immunoblot in *EphA2* KO MSTO-211H cells stably transfected with an empty vector or re-expressing wild-type EphA2, serum-starved for 24 h and exposed to 50 nM progranulin for the indicated time. **D** Parental (P) NCI-H2052 cells, two different *EphA2* KO NCI-H2052 clones and a non-targeting control (NTC) NCI-H2052 clone were serum-starved for 24 h and then stimulated with 50 nM progranulin for 15 min. Levels of total and phosphorylated EphA2, AKT and ERK1/2 were analyzed by immunoblot

We previously demonstrated that EphA2 is the signaling receptor activated by progranulin in bladder cancer [23, 24]. However, the phenotypes of *GRN*- and *EphA2*-deleted cells considerably differed in their migratory, invasive and *in vivo* tumor formation ability, thereby suggesting that EphA2 might not be the major functional progranulin receptor in mesothelioma. Thus, we investigated the impact of EphA2 depletion on the activation of the AKT and MAPK pathways in mesothelioma cells. As shown in Fig. 3A, transient depletion of Epha2 by siRNA strategies did not reduce AKT activation, which was instead clearly affected by transient depletion of progranulin. In addition, the combined depletion of EphA2 and progranulin attenuated the reduction of AKT activation caused by progranulin depletion alone (Fig. 3A). These data demonstrate that progranulin-dependent activation of AKT does not require EphA2. In agreement, MSTO-211H cells with a genetic ablation of *EphA2* showed similar basal pAKT levels when compared to parental MSTO-211H cells (Fig. 3B). In addition, progranulin transient depletion by siRNA led to a reduction in pAKT levels in both parental and *EphA2* KO MSTO-211H cells (Fig. 3B). We then analyzed the effect of progranulin depletion on AKT activation in *EphA2* KO MSTO-211H cells with reconstituted expression of wild type or EphA2 mutants. We expressed wild type EphA2, a kinase-inactive EphA2 mutant (EphA2 K646M) and an EphA2 mutant where the three serine residues at positions 897, 899 and 901, which we identified as phosphorylated upon progranulin stimulation [24], were substituted with alanine residues (EphA2 S897A/S899A/S901A) (Supplementary Fig. 1). Progranulin depletion inhibited AKT activation in all cell lines, further confirming that endogenous progranulin sustains AKT activation in EphA2-independent manner in MSTO-211H cells (Fig. 3B). We then tested whether exogenous progranulin activates AKT and ERK1/2 in MSTO-211H cells lacking EphA2, by comparing *EphA2* KO cells to *EphA2* KO MSTO-211H cells re-expressing wild-type EphA2. Notably, progranulin stimulation triggered AKT and, to a lesser extent, ERK activation independently of the presence of EphA2 (Fig. 3C), further confirming that EphA2 is not required for progranulin-dependent downstream signaling in MSTO-211H. We confirmed these results in parental and *EphA2* KO NCI-H2052 cells further demonstrating that the activation of AKT and MAPK does not require EphA2 (Fig. 3D and and Supplementary Fig. 4D). In summary, these data suggest that progranulin action in mesothelioma cells does not either rely on EphA2 activation or that mesothelioma cells might compensate for the lack of EphA2 by promoting progranulin-dependent AKT and ERK1/2 activation in an EphA2-independent manner.

### Role of Focal adhesion kinase (FAK) in regulating progranulin-dependent activation of AKT and MAPK in mesothelioma

**Fig. 4:**
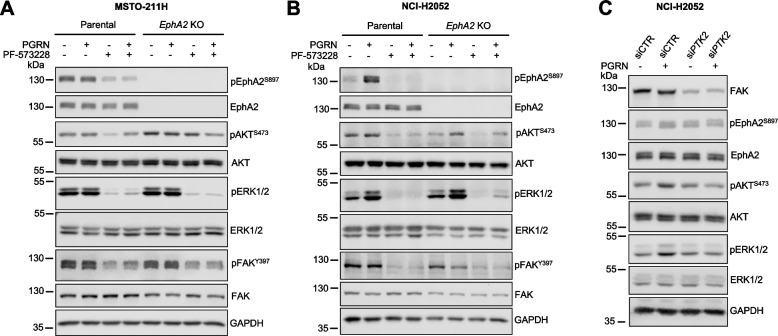
FAK mediates progranulin-dependent activation of AKT and ERK1/2 in mesothelioma cells. **A**, **B** Parental and *EphA2* KO MSTO-211H (**A**) and NCI-H2052 (**B**) cells were serum-starved for 24 h, pre-incubated with the FAK inhibitor PF-573228 (5 µM) for 1 h and then treated with the same concentration of PF-573228 alone or in combination with 50 nM progranulin for 15 min. Levels of total and phosphorylated EphA2 (S897), AKT, ERK1/2 and FAK (Y397) were assessed by immunoblot. **C** NCI-H2052 cells were transfected with siRNA targeting FAK (*PTK2*) or non-targeting control (siCTR) siRNA. At 48 h post-transfection cells were transferred onto serum-free medium and 24 h later exposed to 50 nM progranulin for 15 min. Levels of total and phosphorylated EphA2 (S897), AKT, ERK1/2 and of FAK as assessed by immunoblot

Since it has been previously demonstrated that progranulin modulated focal adhesion kinase (FAK) [18] and FAK plays an important role in modulating growth factors-dependent activation of the AKT and MAPK pathways [40, 41], we investigated the potential role of FAK in mediating progranulin-evoked activation of the AKT and MAPK pathways. Thus, we treated parental, *EphA2* KO MSTO-211H (Fig. 4A) and NCI-H2052 (Fig. 4B) cells with the FAK inhibitor PF-573228 alone or in combination with progranulin. In MSTO-211H cells FAK inhibition led to a significant inhibition of ERK activation, both in basal conditions and upon progranulin-stimulation, and to a partial inhibition of AKT activation in both parental and *EphA2* KO cells (Fig. 4A), suggesting that FAK might have a critical role in controlling ERK activation but only a minor role in modulating AKT signaling in this cell model. In NCI-H2052 cells, both AKT and MAPK pathways were inhibited upon FAK inhibition (Fig. 4B), suggesting a strong dependency of these pathways on FAK activity in this cell model. In agreement, transient knock-down of FAK in NCI-H2052 cells inhibited progranulin-dependent activation of AKT and ERK1/2 (Fig. 4C and Supplementary Fig. 4E). Notably, in both cell lines, FAK inhibition led to a reduction in the levels of phosphorylated EphA2 at S897 (Fig. 4 A-C). Since we demonstrated that EphA2 phosphorylation at S897 mainly relies on ERK1/2 (Fig. 1G), these data suggest that FAK, by controlling ERK1/2 activation, might indirectly sustain EphA2 phosphorylation at S897 in mesothelioma cells.

### Progranulin promotes the activation of multiple RTKs in mesothelioma cells

**Fig. 5:**
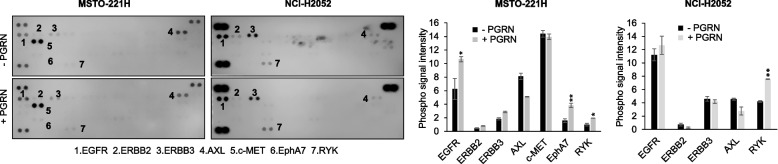
Multiple RTKs are activated by progranulin in mesothelioma cells. **A** Phospo-RTK arrays were treated with lysates from MSTO-211H or NCI-H2052 cells in SFM or treated with 10 nM progranulin for 15 min. Data are mean ± SD, ** *p* < 0.01, * *p* < 0.05

**Fig. 6:**
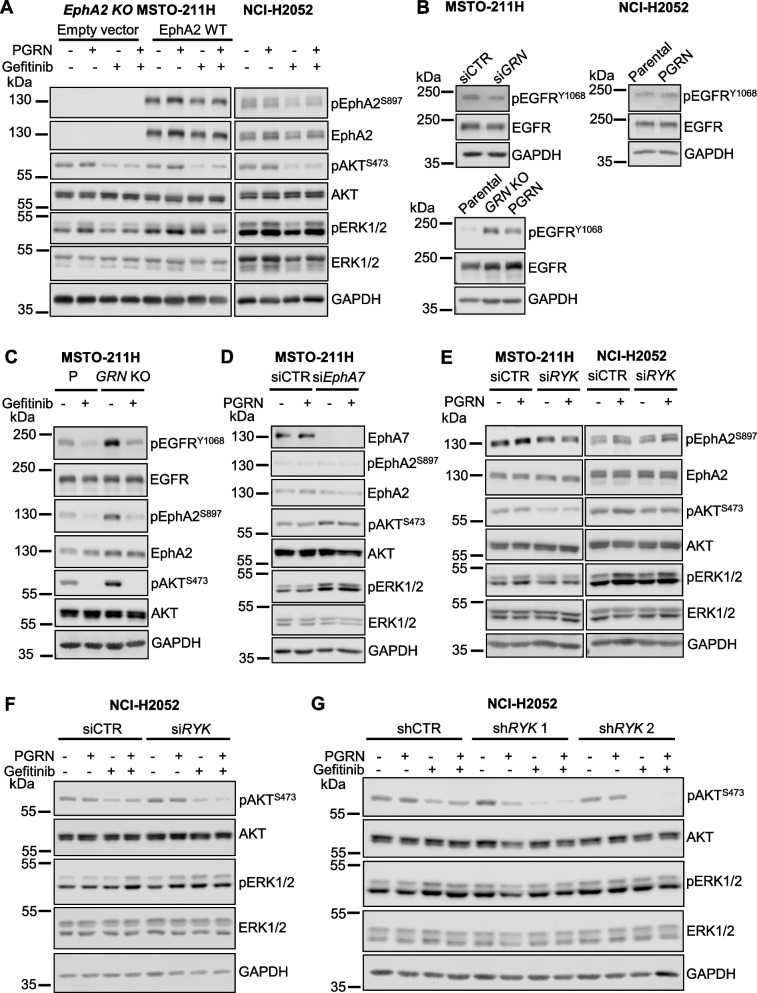
EGFR and RYK sustain progranulin signaling in mesothelioma cells. **A** Total and phosphorylated EphA2 (S897), AKT and ERK1/2 in *EphA2* KO MSTO-211H cells stably transduced with an empty vector or re-expressing wild type EphA2 (left panel) and NCI-H2052 cells (right panel) pre-incubated with the EGFR inhibitor gefitinib (10 µM) for 1 h and then exposed to the same concentration of gefitinib alone or in combination with 50 nM progranulin for 15 min. **B** Total and phosphorylated EGFR (Y1068) in MSTO-211H cells transfected with *GRN*-specific or non-targeting control (siCTR) siRNA (upper left panel), parental and PGRN overexpressing NCI-H2052 cells (upper right panel) and parental, *GRN* KO or PGRN-reconstituted *GRN* KO MSTO-211H cells (lower panel) after serum-starvation for 24 h. **C** Levels of total and phosphorylated EGFR (Y1068), EphA2 (S897) and AKT in parental or *GRN* KO MSTO-211H cells serum-starved for 24 h and treated with gefitinib (10 µM) for 1 h 30 min. **D** Levels of EphA7, total and phosphorylated EphA2 (S897), AKT and ERK1/2 in MSTO-211H cells transfected with *EphA7*-specific or control (siCTR) siRNA, transferred onto serum-free 24 h post transfection, incubated for additional 24 h and then treated with 50 nM progranulin for 15 min. **E** MSTO-211H and NCI-H2052 cells were transfected with siRNA specific for *RYK* or non-targeting control (siCTR) siRNAs. At 48 h post-transfection cells were transferred onto serum-free medium and incubated for 24 h. Cells were then treated with 50 nM PGRN for 15 min. Total and phosphorylated EphA2 (S897), AKT and ERK1/2 were analyzed by immunoblot. **F-G** Total and phosphorylated AKT and ERK1/2 in NCI-H2052 transfected with si*RYK* or control siRNA (siCTR) (**F**) or stably expressing two different *RYK-*specific or a non-targeting control (shCTR) shRNA treated with 10 µM gefitinib for 1 h and then exposed to the same concentration of gefitinib alone or in combination with 50 nM progranulin for 15 min

Contrary to our previous data in bladder cancer [23, 24], our results suggest that EphA2 is not the main functional progranulin signaling receptor in mesothelioma. In addition, recent data have suggested that progranulin might have the ability to activate multiple RTK signaling pathways in cell context-dependent manner [42]. Thus, to identify receptor tyrosine kinases (RTKs) activated by progranulin in mesothelioma cells, we used an unbiased approach by testing antibody arrays that simultaneously assess tyrosine-phosphorylation levels of 49 different human RTKs and exposed these arrays to lysates derived from unstimulated (SFM) or progranulin-stimulated MSTO-211H or NCI-H2052 cells. In MSTO-211H cells, progranulin promoted a significant increase in total tyrosine phosphorylation of EGFR, EphA7 and RYK (Fig. 5, left panels) while in NCI-H2052 cells, progranulin induced a significant increase of RYK tyrosine phosphorylation and a minor increase in EGFR phosphorylation (Fig. 5, right panels). Notably, in these experimental conditions we did not detect tyrosine-phosphorylation of EphA2. Based on these results, we initially investigated the potential role of EGFR in mediating progranulin-dependent activation of AKT and ERK1/2. In MSTO-211H cells, both *EphA2* KO cells and *EphA2* KO cells re-expressing wild type EphA2, pharmacological inhibition of EGFR by the specific, clinical grade, EGFR inhibitor gefitinib reduced pAKT and pERK1/2 levels and prevented progranulin-evoked AKT and ERK1/2 activation (Fig. 6A, left panel), thereby confirming the role of EGFR in mediating progranulin-dependent signaling in MSTO-211H cells. Interestingly, EGFR inhibition led to decreased EphA2 S897 phosphorylation (Fig. 6A, left panel), suggesting that EGFR is at least in part involved in controlling EphA2 serine-phosphorylation in these cells. We performed similar experiments in NCI-H2052 cells where EGFR inhibition was associated with a reduction in the basal levels of pAKT but not pERK1/2 and inhibited progranulin-dependent activation of AKT but not of ERK1/2 (Fig. 6A, right panel), suggesting a more restricted role for EGFR in mediating progranulin-dependent activation of AKT in this cell model. Notably, as in MSTO-211H, EGFR inhibition attenuated the phosphorylation of EphA2 on S897 in NCI-H2052 cells as well (Fig. 6A, right panel), suggesting a modulation of EphA2 activity by EGFR in mesothelioma cells. Based on these results, we investigated whether endogenous progranulin might affect EGFR activation in mesothelioma cells. As shown in Fig. 6B, MSTO-211H cells with transient depletion of progranulin by siRNA strategies showed reduced EGFR phosphorylation at Y1068 when compared to siControl-transfected cells (Fig. 6B, left upper panel, and Supplementary Fig. 4F), indicative of a reduced activation of EGFR. In agreement, progranulin overexpressing NCI-H2052 cells showed slightly increased levels of pEGFR Y1068 when compared to parental NCI-H2052 cells (Fig. 6B, right upper panel, and Supplementary Fig. 4F), suggesting that EGFR activation is dependent on progranulin expression levels. All together these results suggest that progranulin modulates EGFR activation in mesothelioma cells. To confirm these data, we compared the levels of pEGFR Y1068 in parental and *GRN* KO MSTO-211H cells. Notably, we observed higher pEGFR Y1068 phosphorylation levels in *GRN* KO than in parental MSTO-211H cells (Fig. 6B, lower panel, and Supplementary Fig. 4F). These data indicate a complex modulation of EGFR activity by endogenous progranulin and suggest that progranulin genetic deletion might trigger homeostatic compensatory mechanisms leading to enhanced EGFR activation. Considering that EGFR inhibition reduced pEphA2 S897 and pAKT levels (Fig. 6A), we hypothesized that the enhanced pEGFR Y1068 levels in *GRN* KO MSTO-211H cells might determine the increase in pEphA2 S897 and pAKT levels observed in this cell line as compared to parental MSTO-211H cells (Fig. 1F). Indeed, the inhibition of EGFR strongly reduced both pEphA2 S897 and pAKT levels (Fig. 6C), suggesting that the increased activity of EGFR in *GRN* KO MSTO-211H cells is, at least in part, responsible for the increased levels of pEphA2 S897 and pAKT observed in this cell line. Next, we investigated the potential role of EphA7 in regulating progranulin downstream signaling in MSTO-211H cells. Significantly, transient depletion of EphA7 by siRNA approaches increased basal levels of pAKT and pERK1/2 (Fig. 6D), suggesting that EphA7 might have an inhibitory action on AKT and MAPK, ruling out a possible role for EphA7 in mediating progranulin-induced activation of these two signaling pathways. Finally, we investigated the potential role played by RYK in both MSTO-211H and NCI-H2052 by transiently knocking-down RYK using siRNA approaches. Given the very limited availability of reliable antibodies for RYK, we verified the efficiency of RYK knock-down by measuring mRNA levels. As shown in Supplementary Fig. 3A, siRYK-transfected cells showed a significant reduction in RYK mRNA levels (about 80%). In MSTO-211H cells, RYK depletion reduced pAKT basal levels and inhibited progranulin-induced activation of both AKT and ERK1/2, whereas in NCI-H2052 RYK knock-down had only a minor effect on AKT activation without affecting pERK1/2 levels (Fig. 6E and Supplementary Fig. 4G). Thus, we hypothesized that, in NCI-H2052, EGFR might compensate for RYK loss and sustain AKT and ERK activation in RYK-depleted NCI-H2052 cells. To test this hipothesis, we exposed siRYK- and siCTR-transfected NCI-H2052 cells to the EGFR inhibitor gefitinib alone or in combination with progranulin. As shown in Fig. 6F, the combined inhibition of EGFR and RYK was more effective than the inhibition of EGFR alone in reducing the activation of AKT (Fig. 6F, RYK mRNA levels reported in Supplementary Fig. 3B). To confirm these results with a complementary approach, we performed similar experiments in NCI-H2052 cells stably transduced with two different *RYK*-specific shRNAs (Fig. 6G and Supplementary Fig. 4H, RYK mRNA levels reported in Supplementary Fig. 3C) or non-silencing shRNA control. As shown in Fig. 6G, the inhibition of EGFR led to a significantly stronger reduction of pAKT levels in shRYK- than in shCTR-transduced cells, both in basal conditions and upon progranulin stimulation. Taken together, these results suggest that progranulin might sustain the activation of multiple RTKs in mesothelioma cells and signaling triggered by progranulin to AKT and MAPK activation might rely on EGFR and RYK in mesothelioma cells.

### Progranulin modulates FAK activity and focal adhesion turnover

**Fig. 7:**
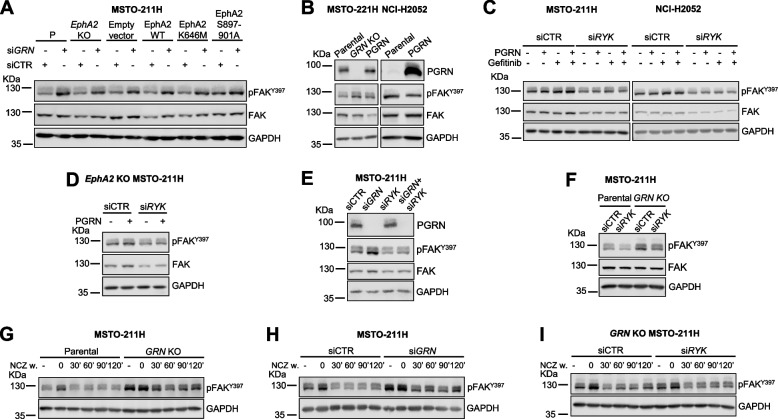
Progranulin modulates pFAK Y397 levels in a RYK-dependent manner and modulates focal adhesion turn over. **A** Parental (P), *EphA2* KO and *EphA2* KO MSTO-211H cells stably transfected with an empty vector, re-expressing wild type EphA2, or EphA2 mutants were transfected with siRNAs targeting progranulin (si*GRN*) or non-targeting (siCTR) control. At 8 h post-transfection cells were serum-starved and total and phosphorylated FAK (Y397) were analyzed by immunoblot 48 h post-transfection. **B** Phosphorylated FAK (Y397) levels in parental, *GRN* KO, *GRN* KO MSTO-211H cells with reconstituted progranulin expression (left panel) or parental and PGRN overexpressing NCI-H2052 cells (right panel) serum-starved for 24 h. **C** Phosphorylated FAK (Y397) in si*RYK*- or siControl-transfected (siCTR) MSTO-211H and NCI-H2052 cells serum-starved for 24 h, pre-incubated with gefitinib (10 µM) for 1 h and then treated with gefitinib alone or in combination with 50 nM progranulin for 15 min. **D** Levels of total and phosphorylated FAK (Y397) in *EphA2* KO MSTO-211H cells transfected with siRNA targeting *RYK* or control (siCTR) siRNA, serum-starved for 24 h and stimulated with 50 nM progranulin for 15 min. **E** Levels of total and phosphorylated FAK (Y397) in MSTO-211H cells transfected with either siRNAs targeting progranulin, *RYK*, their combination or non-targeting controls (siCTR) and serum-starved for 40 h. **F** Levels of total and phosphorylated FAK (Y397) in parental and *GRN* KO MSTO-211H cells transfected with si*RYK* or control (siCTR) siRNA and serum-starved for 24 h. **G-I** Parental and *GRN* KO MSTO-211H cells (**G**), parental MSTO-211H cells 24 h post transfection with siRNA targeting progranulin (si*GRN*) or control ( siCTR) siRNA (H), and *GRN* KO MSTO-211H cells 48 h post transfection with *RYK*-specific siRNA or control (siCTR) siRNA (**I**) were serum-starved for 24 h and then treated with 10 µM nocodazole (NCZ) for 4 h. Nocodazole was then washed-out and the levels of phosphorylated FAK (Y397) were analyzed by immunoblot in cell lysates at the indicated times after nocodazole washout

Because progranulin affected mesothelioma cell motility and adhesion (Fig. 2) and FAK plays a role in mediating progranulin-activated downstream signaling (Fig. 4), we then asked whether progranulin modulation of mesothelioma cell motility might depend on FAK activity. To this end, we first investigated the effect of endogenous progranulin on FAK phosphorylation. We transiently depleted progranulin by siRNA in MSTO-211H cells and observed an increase in pFAK Y397 levels in progranulin-depleted cells as compared to siRNA control-treated cells (Fig. 7A). Notably, progranulin depletion was associated with increased in pFAK Y397 levels in *EphA2* KO MSTO-211H cells and in MSTO-211H cells expressing either wild type, K646M or EphA2 S897A/S899A/S901A EphA2 (Fig. 7A), indicating that the effect of progranulin on pFAK Y397 does not require an active EphA2. To confirm this data, we analyzed by immunoblot pFAK Y397 in *GRN* KO MSTO-211H cells and in *GRN* KO MSTO-211H cells with reconstituted progranulin expression. As shown in Fig. 7B, *GRN* KO cells showed higher pFAK Y397 levels when compared to MSTO-211H parental cells and the reconstitution of progranulin expression partially reduced pFAK Y397 levels observed in *GRN KO* cells (Fig. 7B, left panel, and Supplementary Fig. 4I), confirming the role of progranulin in modulating pFAK Y397 levels. We then analyzed pFAK Y397 levels in NCI-H2052 and observed a slight reduction in FAK phosphorylation at Y397 in cells over-expressing progranulin when compared to parental cells (Fig. 7B, right panel, and Supplementary Fig. 4I), further demonstrating that progranulin modulates FAK phosphorylation in these cells.

Since we have demonstrated that progranulin might activate EGFR and RYK in mesothelioma (Figs. 5 and 6) and considering that both EGFR and RYK might directly or indirectly modulate FAK activity [43–46], we asked whether progranulin-dependent regulation of FAK activity was mediated by EGFR and/or RYK. We therefore depleted RYK by siRNA strategies in MSTO-211H and NCI-H2052 cells (RYK mRNA levels reported in Supplementary Fig. 3D) and exposed both siControl- and siRYK-transfected cells to the EGFR inhibitor gefitinib, progranulin or the combination and analyzed FAK levels. As shown in Fig. 7C, neither cell exposure to the EGFR inhibitor nor cell treatment with progranulin significantly affected pFAK Y397 levels, whereas *RYK* gene knock down led to a reduction of pFAK Y397 levels and to a lesser extent in total levels of FAK in both cell lines (Fig. 7C), pointing out an important role of RYK in modulating FAK activity in mesothelioma. Significantly, similar results were obtained in *EphA2* KO MSTO-211H cells (Fig. 7D, RYK mRNA levels in Supplementary Fig. 3E), suggesting that RYK action on FAK activity does not require EphA2. We then investigated whether the increase in pFAK Y397 levels we observed in MSTO-211H cells upon progranulin depletion was RYK-dependent. Thus, we transiently depleted both progranulin and RYK and observed that RYK knock-down abolished the increase in pFAK Y397 levels associated with progranulin depletion (Fig. 7E and Supplementary Fig. 4J, RYK mRNA levels in Supplementary Fig. 3F). Accordingly, RYK depletion reduced the levels of pFAK Y397 in *GRN* KO MSTO-211H cells (Fig. 7F and Supplementary Fig. 4 K, RYK mRNA levels in Supplementary Fig. 3G*).* Taken together these data suggest that progranulin modulates FAK phosphorylation at Y397 in RYK-dependent fashion.

Since the modulation of FAK phosphorylation at Y397 is associated with focal adhesion (FA) turnover [47], we investigated whether progranulin might affect the kinetics of FAs assembly/disassembly, which is a critical step in the regulation of cell motility [47]. To this end we used a biochemical assay that allows the detailed modulation of FAs turn-over by disrupting the microtubules, which are implicated in FA turnover and cell migration [48–50]. Briefly, cells are first treated with the microtubules-destabilizing drug nocodazole to induce the formation of FAs. Nocodazole is then washed out and the kinetics of FA disassembly and reassembly is evaluated by monitoring the levels of pFAK Y397, as in fact FAK is phosphorylated at Y397 during FA assembly and dephosphorylated upon FA disassembly, which is the essential step for initiating migration [50, 51]. Thus, we exposed parental and *GRN* KO MSTO-211H cells to nocodazole, we then washed it out and analyzed the levels of pFAK Y397 over time. The data shown in Fig. 7G confirmed that *GRN* KO cells have higher basal levels of pFAK Y397 than parental cells. As expected, pFAK Y397 levels increased in both cell lines upon cell treatment with nocodazole, which freezes FAs in the assembled state. After nocodazole release the levels of pFAK Y397 decreased in both cell lines but to a significantly reduced extent in *GRN* KO cells, where pFAK Y397 levels rapidly increased again, indicating a significantly impaired FA disassembly in *GRN* KO cell as compared to parental MSTO-211H cells (Fig. 7G and Supplementary Fig. 4L). Similar results were obtained in parental MSTO-211H cells with transient depletion of progranulin by siRNA (Fig. 7H and Supplementary Fig. 4M). To investigate whether progranulin-dependent modulation of FA turnover might involve RYK, we performed a similar experiment in *GRN* KO MSTO-211H cells transiently transfected with siRNA targeting RYK or controls. As shown in Fig. 7I, RYK transient depletion in *GRN* KO cells showed a persistent reduction in the phosphorylation of FAK Y397 after nocodazole release as compared to siControl-transfected cells (Fig. 7I and Supplementary Fig. 4N, RYK mRNA levels in Supplementary Fig. 3H), suggesting that progranulin-evoked modulation of FA disassembly is regulated by RYK.

**Fig. 8:**
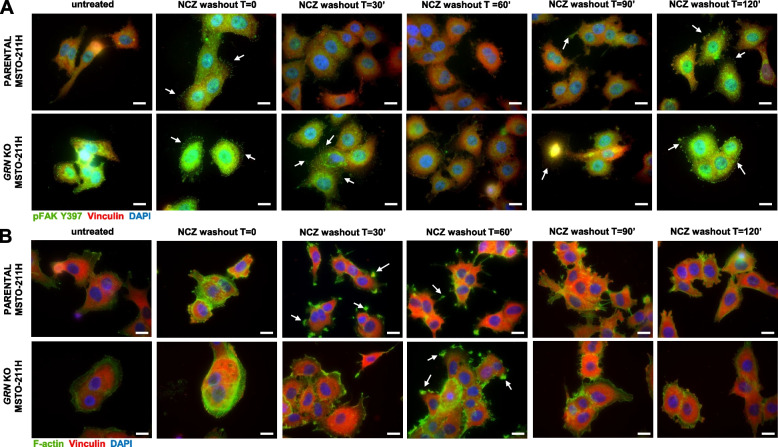
Progranulin modulates focal adhesion turnover and the formation of F-actin-containing cell protrusions. **A-B** Serum-starved parental and *GRN* KO MSTO-211H cells were treated with 10 µM nocodazole (NCZ) for 4 h. Nocodazole was then washed out to allow microtubules regrowth and focal adhesion assembly. Cells were fixed at the indicated time points after nocodazole release and stained for pFAK Y397 and vinculin (**A**) or vinculin and F-actin (**B**) by immunofluorescence. Bars = 20 µm. Arrows indicate focal adhesion (**A**) or F-actin containing cell protrusions (**B**)

We then used a complementary approach and detected FAs by immunofluorescence using pFAK Y397 and vinculin as FAs markers. At 30 min after nocodazole washout FAs could not be detected in parental MSTO-211H cells whereas they could still be detected in *GRN* KO MSTO-211H cells (Fig. 8A, arrows), suggesting that FAs completely disassembled in MSTO-211H parental cells whereas they only partially disassembled in cells lacking progranulin. At 60 min after nocodazole washout FAs could not be detected in both cell lines (Fig. 8A). Collectively these data suggest that cells lacking progranulin have a delayed FAs disassembly. At 90 min from nocodazole release FAs started reforming and they could be detected again in both parental and *GRN* KO MSTO-211H KO cells 2 h post nocodazole release (Fig. 8A, arrows). We then monitored the formation of F-actin-containing cell protrusions in the same experimental conditions. As shown in Fig. 8B, F-actin-containing cell protrusions were already visible at 30 min after nocodazole wash out in parental cells, whereas in *GRN* KO MSTO-211H cells they were detectable at the later time point of 60 min (Fig. 8B, arrows). All together these data suggest that cells lacking progranulin have different kinetics of FAs turnover and cytoskeleton rearrangements, suggesting that progranulin modulates mesothelioma cell motility by affecting FAs disassembly and the formation of cellular protrusions.

## Discussion

Mesothelioma is a rare aggressive malignancy with limited therapeutic options and the response to currently available therapies is highly dependent on tumor histologic subtype [7]. Progranulin is a pleiotropic growth factor playing a critical role in cell proliferation, angiogenesis, and development [34, 52]. Progranulin is often dysregulated in cancer, where it affects both tumor initiation and progression [53]. Very little is currently known about the role of progranulin in mesothelioma but data suggest a role of progranulin in regulating angiogenesis in a VEGF-independent manner [30]. Here, we demonstrate that: 1) progranulin is expressed at high levels in mesothelioma cell lines representative of various mesothelioma histopathological subtypes as compared to non-transformed mesothelial cells; 2) progranulin sustains the activation of the AKT and MAPK signaling pathways in mesothelioma cells, with progranulin signaling being mediated by EGFR, RYK and FAK; 3) progranulin modulates mesothelioma cell migration, invasion, adhesion and *in vivo* tumor growth; 4) progranulin regulates focal adhesion turnover by affecting FAK activation in a RYK-dependent manner. Thus, our results suggest a complex and critical role for progranulin in modulating mesothelioma cell transformation.

Although progranulin oncogenic role has been demonstrated in several tumor types, how progranulin exerts its activity is not fully defined. Indeed, progranulin mechanism of action might rely on its ability to interact with multiple proteins, including component of the extracellular matrix, cell membrane receptors and non-receptor proteins, lysosomal enzymes and trafficking proteins [52]. Our group has recently demonstrated that in bladder cancer progranulin oncogenic activity relies on EphA2, which is the progranulin functional receptor in this tumor model [23, 54]. In bladder cancer, EphA2 activation by progranulin mediates progranulin-dependent activation of AKT and MAPK pathways, thereby sustaining EphA2 phosphorylation at S897 [24]. Here, we found that pEphA2 S897 levels were higher in mesothelioma cells than normal mesothelial cells and that EphA2 phosphorylation at S897 was sustained by progranulin stimulation in an ERK1/2-dependent manner. However, EphA2 is not the main receptor of progranulin in this system as in fact *EphA2* deletion by CRISPR/Cas9 approaches did not prevent progranulin-dependent activation of AKT and MAPK pathways. More importantly, the phenotypes of *GRN*- and *EphA2*-deleted cells were very different in terms of cell migration, invasion, adhesion and *in vivo* tumor formation. In addition, the biological function of progranulin and EphA2 differed in the two cell line models MSTO-211H and NCI-H2052. All together, these results suggest that EphA2 is not the main mediator of progranulin action in mesothelioma cells and that the role of progranulin and EphA2 in mesothelioma is context dependent and might depend on the specific mesothelioma tumor subtype. In addition, EphA2 is phosphorylated at S897 in mesothelioma cells but the relevance of this event is not clear at the moment. In order to explain the different effects of progranulin on cell motility of MSTO-211H and NCI-H2052 cells, we should consider that progranulin can be processed by different proteases into smaller granulins, which often have opposing biological functions [55, 56]. Thus, we can hypothesize that, in NCI-H2052, progranulin could be processed into granulins with an inhibitory effect on cell migration. Further experiments are required to fully elucidate these differences, which might also depend on the particular MM subtype from which cells were derived.

In agreement with the results obtained in *EphA2* KO cells pointing out that EphA2 is not the principal progranulin receptor in mesothelioma, in phospho-RTK arrays-based experiments we did not detect any increase in EphA2 tyrosine phosphorylation in mesothelioma cells stimulated with progranulin, indicating that EphA2 is not directly activated by progranulin in this model. By contrast, the phospho-RTK array data suggested that EGFR, RYK and EphA7 are activated upon progranulin stimulation of mesothelioma cells. Thus, these results suggest that progranulin has an important role in sustaining the activity of RTKs important for the establishment and maintenance of mesothelioma malignant phenotype. As mentioned above, EphA2 is the key mediator of progranulin signaling in bladder cancer where progranulin did also modestly activate EGFR, EphA4 and EphB2 [23]. In mammary epithelial cells multiple RTKs are activated by progranulin, including EGFR, ERBB2 and members of the Eph family [42]. In the neuron-like cell line NSC-34, progranulin also promoted the activation of RET [42]. Thus, our results further support the notion that progranulin might activate multiple RTKs and that progranulin downstream oncogenic signaling might be cell context-dependent.

The modulation of EGFR activity by progranulin was of particular interest. EGFR inhibition affected progranulin-dependent activation of AKT and MAPK and transient progranulin depletion led to a reduction in the levels of pEGFR Y1068 in MSTO-211H cells. However, genetic ablation of progranulin in MSTO-211H cells enhanced both total EGFR and pEGFR Y1068 levels in *GRN* KO cells. The increase in EGFR levels and activity might represent a homeostatic compensatory mechanism for progranulin loss in mesothelioma, which is reminiscent of the increase in expression of several neurotrophic receptors, including RTKs, molecules of the semaphorin-signaling pathway, Notch-related receptors and receptors belonging to the WNT signaling pathways, observed in progranulin-depleted neuronal cells [42]. Notably, the increase of EGFR activity detectable in *GRN* KO cells might explain the enhanced pAKT and pEphA2 S897 levels observed in this cell line as compared to parental cells. Indeed, we showed that in MSTO-211H cells EGFR modulates both AKT and EphA2 S897 phosphorylation and that the inhibition of EGFR in *GRN* KO cells strongly reduced the levels of both EphA2 S897 and pAKT. Importantly, the increase in both EGFR and AKT activity in *GRN* KO cells might explain the paradoxical behavior shown by these cells *in vivo*, as in fact *GRN* KO cells formed larger tumors than parental cells, in spite of reduced motility and invasive ability. This result was somewhat unexpected but not totally surprising, as these data are similar to previously published work on *ERK5* oncogene deletion, which accelerated *in vivo* tumor growth while inhibiting tumor invasion of breast cancer cells [57].

These results should be taken into account when designing potential therapeutic strategies targeting progranulin, as in fact they suggest the need for combinatorial treatments targeting not only progranulin but also the compensatory molecular pathways activated upon progranulin deletion. However, it is important to keep in mind that pharmacological targeting of progranulin might have an effect different from progranulin genetic deletion.

Interestingly, our results provide the first evidence that progranulin might modulate RYK activity. RYK is considered a receptor or coreceptor for the WNT signaling pathway [58, 59] and can mediate both *canonical* and *non-canonical* WNT signaling [60], even though the molecular mechanisms of action are not clearly defined. Indeed, RYK does not appear to have intrinsic kinase activity [61] and it might actually work by functionally interacting with intracellular proteins such as c-SRC or membrane receptors including DZ and Eph receptors [62, 63]. RYK signaling in cancer is highly context dependent. In gastric cancer and glioma, RYK promotes cell migration and invasion [64–66] whereas in prostate cancer Wnt5a-RYK have pro-apoptotic and pro-proliferative action [67]. In lung cancer, RYK promotes resistance acquired upon EGFR inhibition [68]. In mesothelioma, the WNT pathway controls cell proliferation, apoptosis and cisplatin-resistance [69–71] and RYK phosphorylation was observed in 7 out of 12 diffuse malignant peritoneal mesothelioma frozen samples [72]. However, the role of RYK in mesothelioma cells remains unexplored. In addition, there are no reports suggesting a role of progranulin in directly modulating the WNT pathway in cancer, even though recent data have demonstrated that progranulin depletion might increase the expression of some WNT pathways receptors [42]. In addition, *GRN* haploinsufficiency detectable in patients with frontotemporal lobar degeneration is associated with Wnt5a signaling in neuronal cells but no connection with RYK has been established [73, 74]. Thus, whether progranulin might directly interact with RYK, thereby competing with WNT proteins for RYK binding, is currently not established. Notably, it has been reported a role of RYK in modulating PI3K/AKT pathway [75]. Accordingly, our results demonstrate RYK action in modulating AKT activation in mesothelioma, as in fact RYK depletion inhibited basal and progranulin-dependent AKT activity.

Interestingly, both EGFR and RYK inhibition led to a reduction of pEphA2 S897 levels, suggesting that EphA2 S897 phosphorylation observed upon progranulin stimulation might not be direct but secondary to EGFR and RYK activation. The crosstalk between EphA2 and EGFR is well documented and this functional interaction is quite complex and context-dependent being both protumorigenic [76] and anti-migratory, by inhibiting EGF-modulated AKT activation [77]. Similarly, there are reports suggesting that Eph receptors, including EphA2, might cross-talk with RYK and promote RYK phosphorylation [63]. Previous data have demonstrated that several RTKs are coactivated in mesothelioma cells and sustain the activity of the AKT and MAPK pathways, suggesting that the combined inhibition of multiple RTKs would be the best strategy to counteract their pro-tumorigenic action [72, 78]. Our results highlight the complexity of progranulin signaling in mesothelioma and suggest that progranulin might be a growth factor able to sustain the activation of multiple RTKs, thereby modulating their crosstalk and downstream activation of AKT and MAPK signaling. The picture is even more complex if we consider that the results of the phospho-RTK arrays suggested that EphA7 might be also tyrosine-phosphorylated upon progranulin stimulation. The role of EphA7 in cancer is very controversial and context-dependent, with reports suggesting both pro-malignant and anti-tumorigenic function [63]. Our data suggest that EphA7 might have a tumor suppressive role in mesothelioma, since EphA7 depletion did not prevent progranulin-dependent AKT and MAPK activation but led instead to an increase in basal activation of these two signaling pathways.

FAK is a key regulator of mesothelioma cell proliferation, survival, migration, invasion, adhesion and maintenance of cancer stem cells (CSC) and is becoming a very attractive target for cancer therapy [79–81]. Mesothelioma cells lacking the expression of the tumor suppressor neurofibromatosis type 2 (*NF2*) gene, merlin, show strong dependency on FAK signaling [82, 83]. *NF2* mutations are among the most common genetic alterations in mesothelioma, with *NF2* biallelic loss being present in about 40% to 50% of tumors [83]. Merlin is expressed at the cell–cell boundary where it controls the maturation of adherens junctions. Cells lacking merlin have weaker cell–cell adhesion, rely on FAK-evoked survival signals coming from the extracellular matrix [83], show altered RTKs trafficking [84] and present enhanced Ras-MEK1/2-ERK1/2 signaling [82]. Based on these published data, we can therefore speculate that the presence or absence of merlin in mesothelioma cells might determine different EGFR/RYK endocytosis/sorting, which could modulate the intensity of progranulin-dependent downstream signaling.

This study highlights a complex modulation of FAK activity by progranulin. Indeed, FAK inhibition had a strong inhibitory effect on ERK1/2 activation and abolished progranulin-dependent activation of ERK1/2 independently of EphA2, in both MSTO-211H and NCI-H2052 cells, demonstrating the role for FAK in mediating progranulin-dependent activation of MAPK in mesothelioma. In addition, we showed that FAK modulated EphA2 phosphorylation at S897. This aspect might be relevant for NCI-H2052 cells, where activated EphA2 might partially control cell migration. In addition, it might have a role in MSTO-211H cell adhesion to fibronectin considering that *EphA2* KO MSTO-211H cells showed an increased adhesion to fibronectin when compared to parental cells. Notably, an interplay between EphA2, FAK and fibronectin has been recently reported [85]. Finally, in NCI-H2052 cells (merlin-negative), FAK inhibition had a strong inhibitory effect on AKT activation, both basal and progranulin-dependent, whereas the effect was only minor in MSTO-211H cells (merlin-positive). These data are in agreement with the results reported by Shapiro et al. [83], who demonstrated that the modulation of AKT by FAK is affected by merlin expression in mesothelioma cells. Considering the relevance of FAK and AKT in promoting cell migration and adhesion, we can therefore speculate that the difference in progranulin-evoked migratory and adhesive response between MSTO-211H and NCI-H2052 cells could be dependent on the presence or absence of merlin, which might be critical for FAK activity. We additionally demonstrated that progranulin depletion in MSTO-211H cells led to significant increase in FAK Y397 phosphorylation. Interestingly, this effect was reverted by RYK depletion, suggesting that this process is RYK-dependent, thereby pointing out a complex interplay between progranulin, FAK and RYK. Of note, RYK depletion led to reduced levels of pFAK Y397 in both MSTO-211H and NCI-H2052, suggesting a novel role for RYK in modulating FAK activity. A functional cross-talk between WNT and FAK pathways has been previously reported in several cancer models [45, 46], but there are no data supporting a role of RYK in this process. Given the key role of FAK in mesothelioma, especially in merlin-negative cells, it would be interesting to investigate whether modulating RYK expression/activity might influence mesothelioma cell motility and adhesion and/or the proliferation and survival of mesothelioma CSCs derived from *NF2*^*−/−*^ tumors. Progranulin depletion strongly enhances FAK phosphorylation at Y397, site critical for FA turnover. Notably, FAK Y397 phosphorylation promotes FA assembly, followed by FAK Y397 dephosphorylation and FA disassembly, which is the fundamental step required for cell motility [47, 50, 51, 86]. Indeed, FAK^−/−^ fibroblasts had an increased number of FA, reduced FA disassembly and reduced migration [87]. It has been demonstrated that FA disassembly relies on the targeting of FA by microtubules and on different molecules including dynamin, whose localization and activity at the site of FA is regulated by FAK [50, 88]. Here we demonstrated that mesothelioma cells with progranulin depletion have altered FA turnover, with slower and reduced FA disassembly. Thus, the inhibition of FA disassembly in progranulin-depleted mesothelioma cells is likely the biochemical mechanism determining the reduced migratory and invasive abilities of these cells.

## Conclusion

Our results point out to a complex progranulin signaling mechanisms in mesothelioma, where progranulin mediates the activation and cross-talk of multiple RTKs with key roles in establishing and maintaining mesothelioma malignant phenotypes, where progranulin signaling is context-dependent. Our study suggests that blocking progranulin signaling might represent a viable therapeutic strategy for mesothelioma. However, the efficacy of potential therapeutic strategies targeting progranulin might differ depending on mesothelioma subtype and combinatorial approaches inhibiting homeostatic compensatory mechanisms might be required.

## Supplementary Information

**Additional file 1: Supplementary Fig. 1.** *EphA2 KO* mesothelioma cell lines and mesothelioma cell lines expressing EphA2 mutants. **A** Levels of total and phosphorylated (S897) EphA2 in parental and *EphA2* KO MSTO-211H cells and in *EphA2* KO MSTO-211H cells transduced with an empty vector or a vector containing the cDNA coding for wild type, K646M or S897A/S899A/S901A EphA2 mutants. **B** Levels of EphA2 in parental NCI-H2052 cells, two different clones of NCI-H2052 *EphA2* KO cells generated by CRISPR/Cas9 approach, and cells expressing a non-targeting guide RNA.

**Additional file 2: Supplementary Fig. 2.** NCI-H2052 adhesion to plasma fibronectin, collagen, poly-L-Lys and cell-cell adhesion.** A-B** The ability of parental, progranulin overexpressing and *EphA2* KO NCI-H2052 cells to adhere to plasma fibronectin, collagen and poly-L-Lys (A) or adhere to a monolayer of the indicated cell lines (B) was assessed as described in Material and Methods.

**Additional file 3: Supplementary Fig. 3.** RYK mRNA levels in siRNA experiments performed. To assess the efficiency of *RYK* targeting by siRNA or shRNA, mRNA levels of RYK were assessed by qPCR in cells used for the experiments shown in the following Figures: A) Fig. 6E; B) Fig. 6F; C) Fig. 6G; D) Fig. 7C; E) Fig. 7D; F); Fig. 7E; G) Fig. 7F; H) Fig. 7I. β-actin was used as housekeeping gene.

**Additional file 4: Supplementary Fig. 4.** Immunoblots quantifications. Quantifications of immunoblot panels shown in the following Figures: A) Fig. 1D; B) Fig. 1E; C) Fig. 1F; D) Fig. 3D; E) Fig. 4C; F) Fig. 6B; G) Fig. 6E; H) Fig. 6G; I) Fig. 7B; J) Fig. 7E; K) Fig. 7F; L) Fig. 7G; M) Fig. 7H; N) Fig. 7I.

**Additional file 5: Supplementary table 1.** Nucleotide sequence of the primers used in the study.

## Data Availability

All data generated or analyzed during this study are included in this published article and its supplementary information files.

## Abbreviations

EGFR: Epidermal growth factor receptor
EphA2: Erythropoietin-producing hepatocellular carcinoma receptor A2
ERK: Extracellular signal-regulated kinase
FA: Focal adhesion
FAK: Focal adhesion kinase
FN: Fibronectin
MAPK: Mitogen-activated protein kinase
MM: Malignant mesothelioma
NF2: Neurofibromatosis type 2
PGRN: Progranulin
PTK2: Protein tyrosine kinase 2
RTK: Receptor tyrosine kinase
RYK: Receptor-like tyrosine kinase
VEGF: Vascular endothelial growth factor
WNT: Wingless/integrated
