# Supplementary figures and images for: Complexity of progranulin mechanisms of action in mesothelioma
Source: J Exp Clin Cancer Res. 2022 Dec 5;41:333. doi: 10.1186/s13046-022-02546-4 (PMC9720952; doi:10.1186/s13046-022-02546-4)

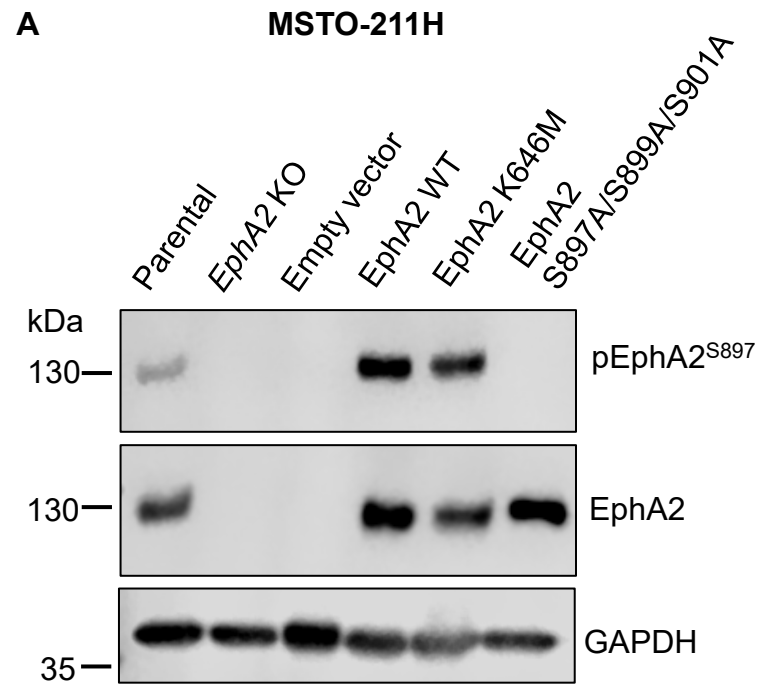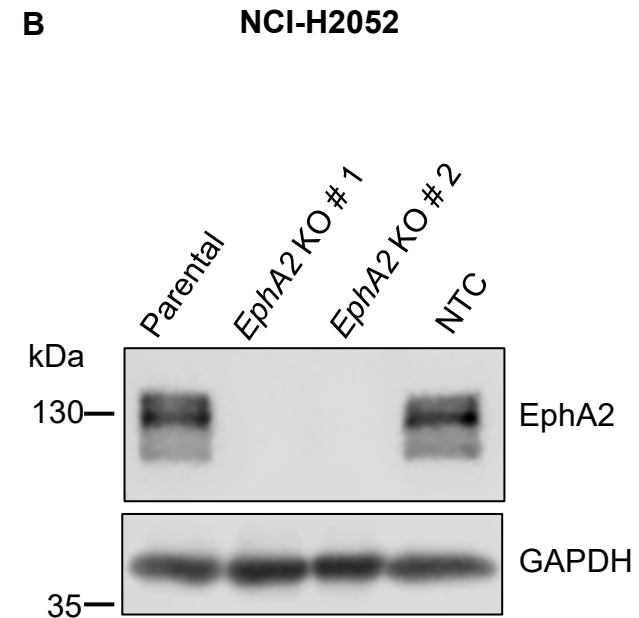

Supplement: Supplementary file 1 — Additional file 1: Supplementary Fig. 1. EphA2 KO mesothelioma cell lines and mesothelioma cell lines expressing EphA2 mutants. A Levels of total and phosphorylated (S897) EphA2 in parental and EphA2 KO MSTO-211H cells and in EphA2 KO MSTO-211H cells transduced with an empty vector or a vector containing the cDNA coding for wild type, K646M or S897A/S899A/S901A EphA2 mutants. B Levels of EphA2 in parental NCI-H2052 cells, two different clones of NCI-H2052 EphA2 KO cells generated by CRISPR/Cas9 approach, and cells expressing a non-targeting guide RNA. [file 13046_2022_2546_MOESM1_ESM.pdf]

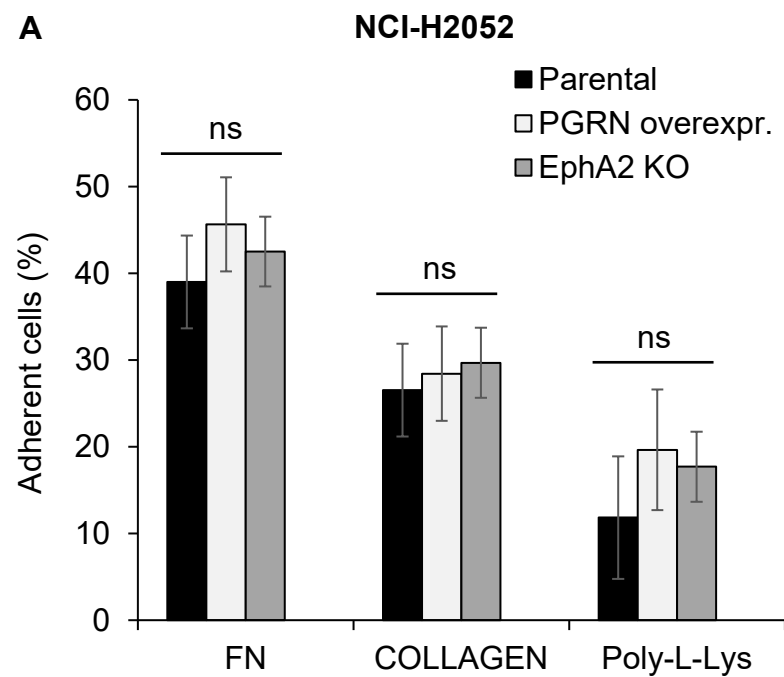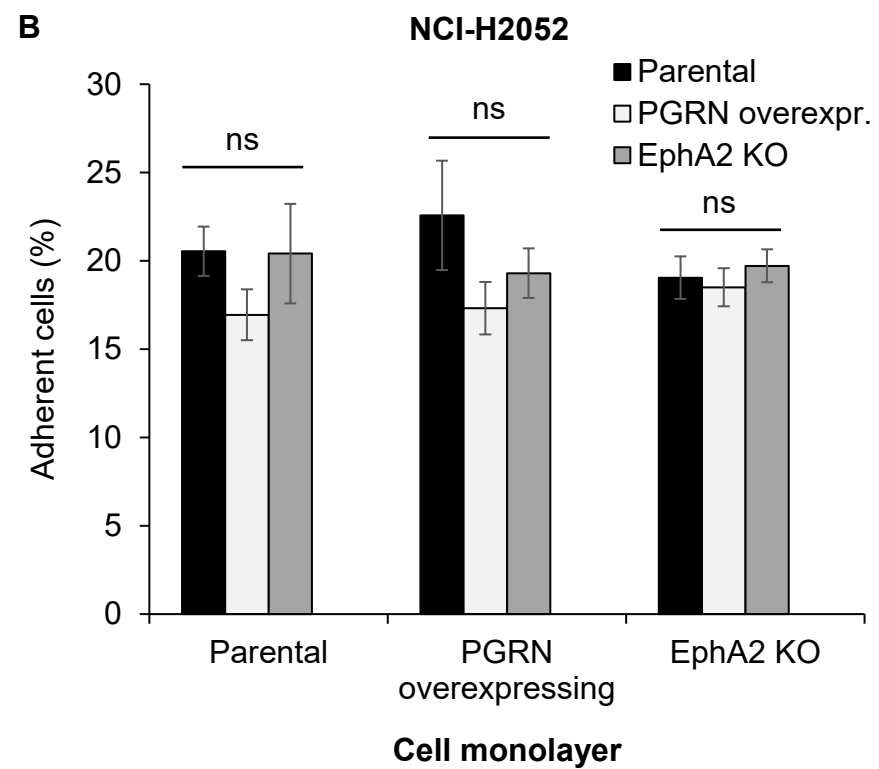

Supplement: Supplementary file 2 — Additional file 2: Supplementary Fig. 2. NCI-H2052 adhesion to plasma fibronectin, collagen, poly-L-Lys and cell-cell adhesion. A-B The ability of parental, progranulin overexpressing and EphA2 KO NCI-H2052 cells to adhere to plasma fibronectin, collagen and poly-L-Lys (A) or adhere to a monolayer of the indicated cell lines (B) was assessed as described in Material and Methods. [file 13046_2022_2546_MOESM2_ESM.pdf]

**A**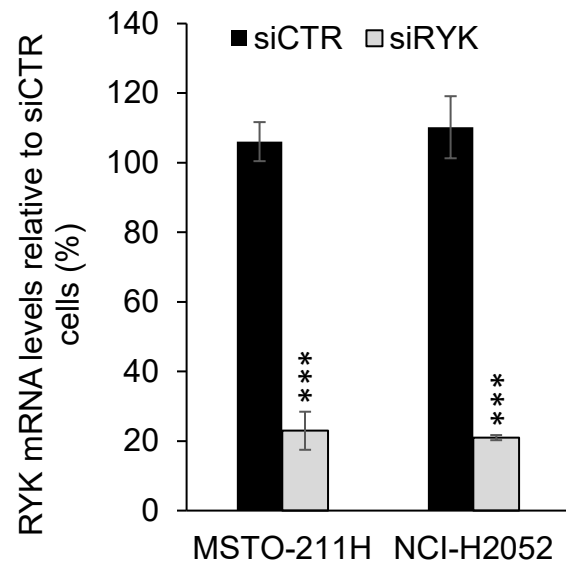**B**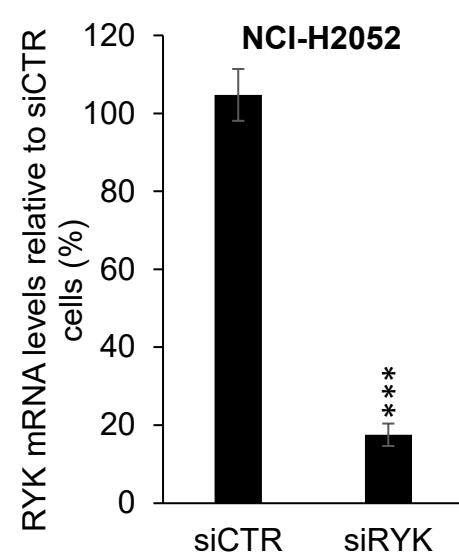**C**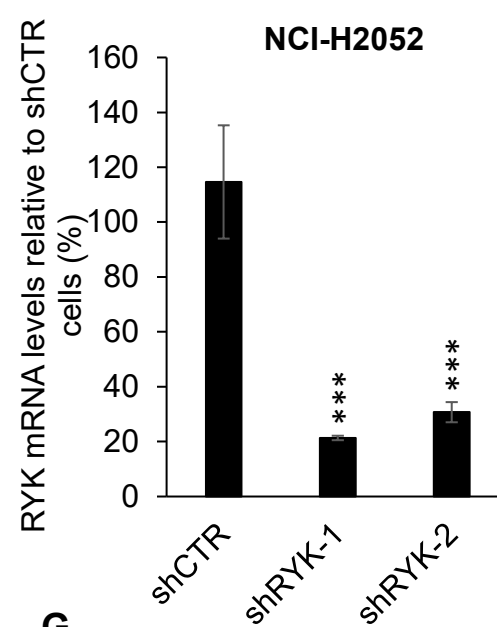**D**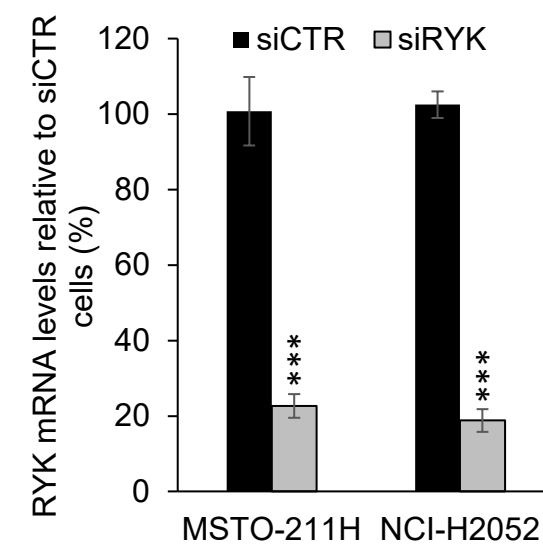**E**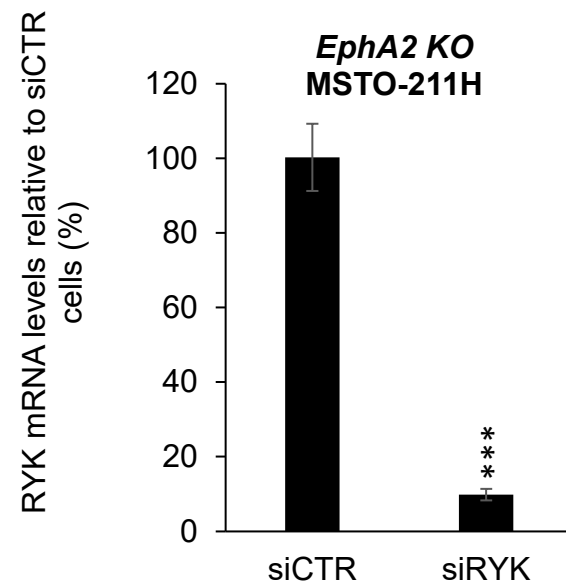**F**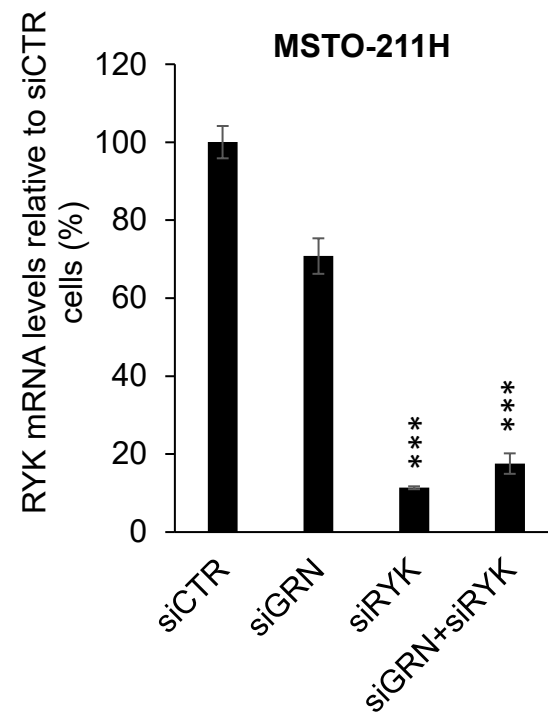**G**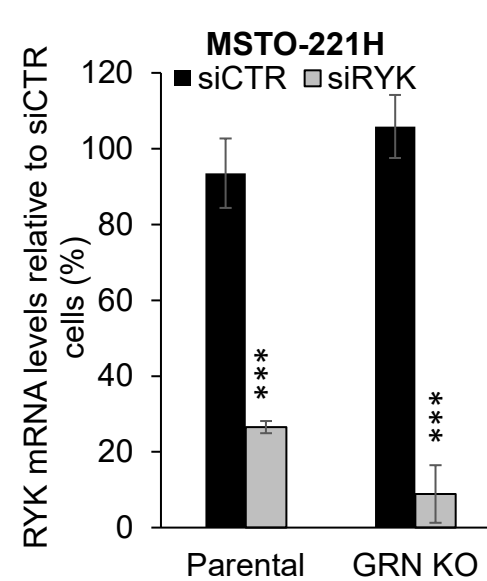**H**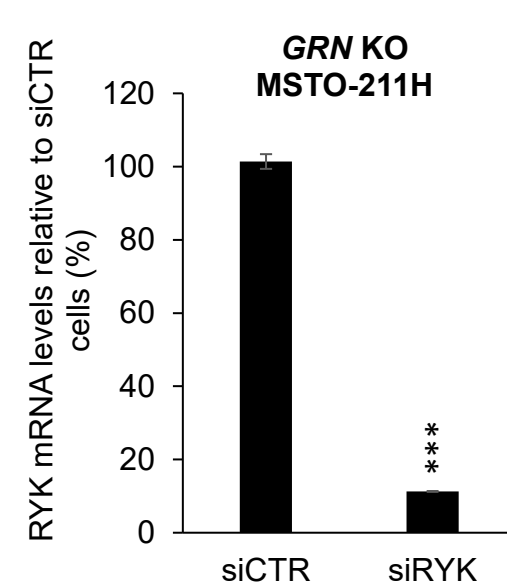**Supplementary Fig. 3**

Supplement: Supplementary file 3 — Additional file 3: Supplementary Fig. 3. RYK mRNA levels in siRNA experiments performed. To assess the efficiency of RYK targeting by siRNA or shRNA, mRNA levels of RYK were assessed by qPCR in cells used for the experiments shown in the following Figures: A) Fig. 6E; B) Fig. 6F; C) Fig. 6G; D) Fig. 7C; E) Fig. 7D; F); Fig. 7E; G) Fig. 7F; H) Fig. 7I. β-actin was used as housekeeping gene. [file 13046_2022_2546_MOESM3_ESM.pdf]

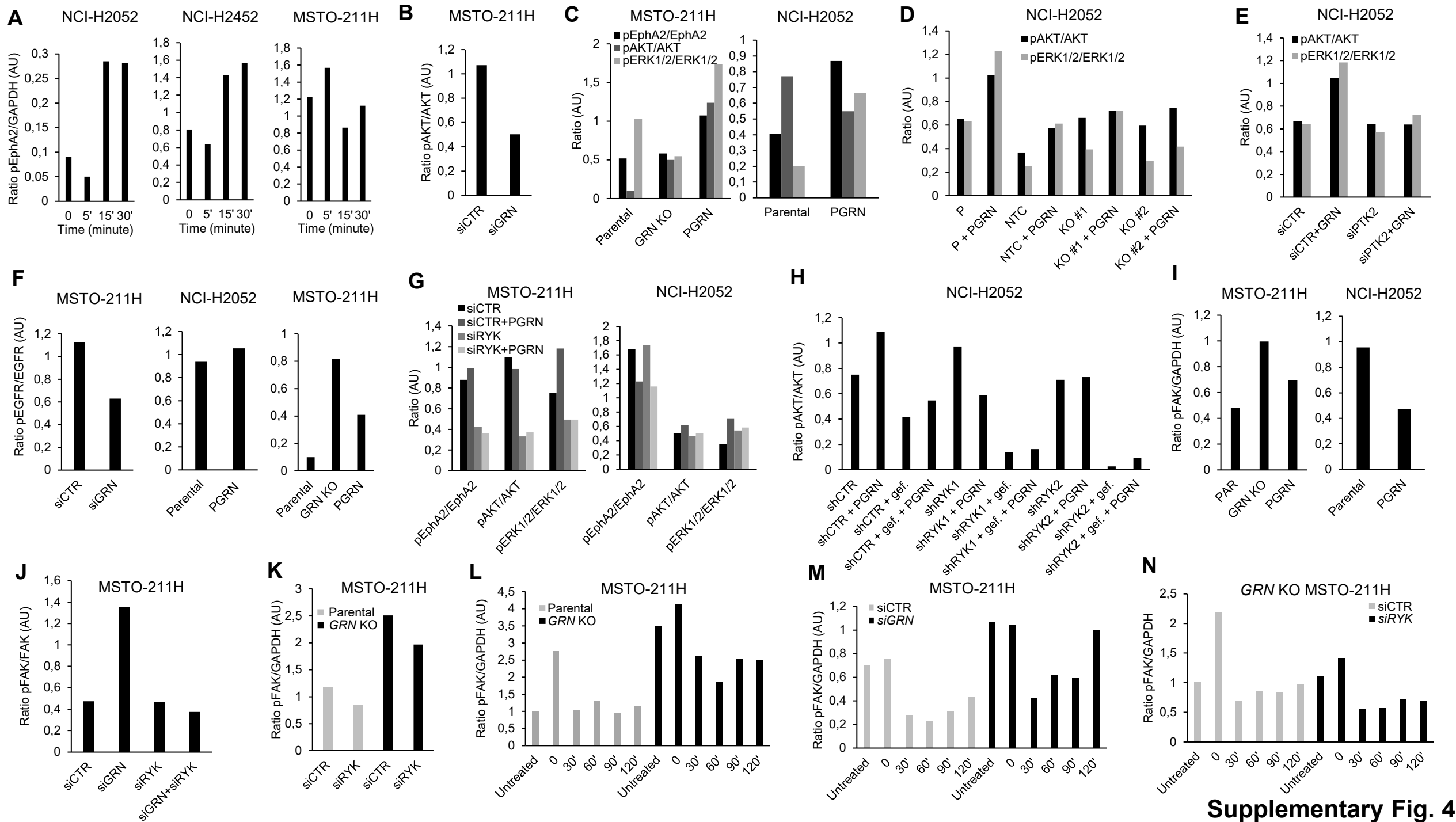

**Supplementary Fig. 4**

Supplement: Supplementary file 4 — Additional file 4: Supplementary Fig. 4. Immunoblots quantifications. Quantifications of immunoblot panels shown in the following Figures: A) Fig. 1D; B) Fig. 1E; C) Fig. 1F; D) Fig. 3D; E) Fig. 4C; F) Fig. 6B; G) Fig. 6E; H) Fig. 6G; I) Fig. 7B; J) Fig. 7E; K) Fig. 7F; L) Fig. 7G; M) Fig. 7H; N) Fig. 7I. [file 13046_2022_2546_MOESM4_ESM.pdf]
