# Supplementary material for: Complexity of progranulin mechanisms of action in mesothelioma
Source: J Exp Clin Cancer Res. 2022 Dec 5;41:333. doi: 10.1186/s13046-022-02546-4 (PMC9720952; doi:10.1186/s13046-022-02546-4)
Supplement: Supplementary file 5 — Additional file 5: Supplementary table 1. Nucleotide sequence of the primers used in the study. [file 13046_2022_2546_MOESM5_ESM.pdf]

Primer name	Primer sequence
GRN fwd	TGGACCCTGGTGAGCTGG
GRN rev	CAGCAGCTGTCTCAAGGCTGGG
NcoI *GRN* fwd	AATTCACCATGGCATGGACCCTGGTGAGCTGG
NotI *GRN* rev	CTCGCGGCCGCTTATCACAGCAGCTGTCTCAAGGCTGGG
XhoI EphA2 fwd	GTTAACTCGAGATGGAGCTCCAGGC
BamHI EphA2 rev	GATGGGGATCCCCACAGTGTTCACCTGGTCC
EphA2 K646M fwd	CCGGTGGCCATCATGACGCTGAAAGCC
EphA2 K646M rev	GGCTTTCAGCGTCATGATGGCCACCGG
EphA2 S897A/S899A/S901A fwd	CTATCCGGCTCCCCGCCACGGCCGGCGCCGAGGGGGTGCC
EphA2 Ser cluster rev	GGGGAGCCGGATAGACACGCG
RYK fwd	CAACTCCTATCACCAGCTCCTT
RYK rev	TTGGCCTCCAAAAGAGTGACA
β-actin fwd	CAGGGCGTGATGGTGGGC
β-actin rev	CTCGGTCAGCAGCACGG

Supplementary Table 1. Nucleotide sequence of the primers used in the study.
